# BAMBI Is Expressed in Endothelial Cells and Is Regulated by Lysosomal/Autolysosomal Degradation

**DOI:** 10.1371/journal.pone.0012995

**Published:** 2010-09-24

**Authors:** Sandhya Xavier, Victoria Gilbert, Maria Pia Rastaldi, Stefanie Krick, Dmitrij Kollins, Anand Reddy, Erwin Bottinger, Clemens D. Cohen, Detlef Schlondorff

**Affiliations:** 1 Department of Medicine, Mount Sinai School of Medicine, New York, New York, United States of America; 2 Division of Nephrology and Institute of Physiology with Center of Integrative Human Physiology, University Hospital and University of Zurich, Zurich, Switzerland; 3 Renal Immunopathology Laboratory, Fondazione D'Amico per la Ricerca sulle Malattie Renali, Milan, Italy; Indiana University, United States of America

## Abstract

**Background:**

BAMBI (**B**MP and **A**ctivin **M**embrane **B**ound **I**nhibitor) is considered to influence TGFβ and Wnt signaling, and thereby fibrosis. Surprisingly data on cell type-specific expression of BAMBI are not available. We therefore examined the localization, gene regulation, and protein turnover of BAMBI in kidneys.

**Methodology/Principal Findings:**

By immunofluorescence microscopy and by mRNA expression, BAMBI is restricted to endothelial cells of the glomerular and some peritubular capillaries and of arteries and veins in both murine and human kidneys. TGFβ upregulated mRNA of BAMBI in murine glomerular endothelial cells (mGEC). LPS did not downregulate mRNA for BAMBI in mGEC or in HUVECs. BAMBI mRNA had a half-life of only 60 minutes and was stabilized by cycloheximide, indicating post-transcriptional regulation due to AU-rich elements, which we identified in the 3′ untranslated sequence of both the human and murine BAMBI gene. BAMBI protein turnover was studied in HUVECs with BAMBI overexpression using a lentiviral system. Serum starvation as an inducer of autophagy caused marked BAMBI degradation, which could be totally prevented by inhibition of lysosomal and autolysosomal degradation with bafilomycin, and partially by inhibition of autophagy with 3-methyladenine, but not by proteasomal inhibitors. Rapamycin activates autophagy by inhibiting TOR, and resulted in BAMBI protein degradation. Both serum starvation and rapamycin increased the conversion of the autophagy marker LC3 from LC3-I to LC3-II and also enhanced co-staining for BAMBI and LC3 in autolysosomal vesicles.

**Conclusions/Significance:**

1. BAMBI localizes to endothelial cells in the kidney and to HUVECs. 2. BAMBI mRNA is regulated by post-transcriptional mechanisms. 3. BAMBI protein is regulated by lysosomal and autolysosomal degradation. The endothelial localization and the quick turnover of BAMBI may indicate novel, yet to be defined functions of this modulator for TGFβ and Wnt protein actions in the renal vascular endothelium in health and disease.

## Introduction

BAMBI (BMP and Activin receptor Membrane Bound Inhibitor) was cloned in 1999 and shown to interact and inhibit the TGFβ type I receptor and TGFβ signaling in a xenopus system and in mouse embryonic carcinoma P19 cells through the SMAD 2 and 3 dependent pathway [1]. Based on these interactions the protein was called “BMP and Activin receptor Membrane Bound Inhibitor (BAMBI)” and was proposed to function as a competitive receptor antagonist for the TGFβ receptor family and the subsequent Smad signaling pathways [2]. Recently a role for BAMBI in the Wnt/β-catenin signaling pathway has also been postulated [3], [4]. Lin et al (2008) demonstrated that BAMBI interacts with Wnt receptor Fzd, the co-receptor LRP6, as well as Dvl2, and promotes Wnt-induced cell cycle progression and proliferation. That BAMBI may be involved with cell proliferation was suggested by numerous observations, where BAMBI was found to be overexpressed in tumors [3]. In the tumors the expression of BAMBI seemed to correlate with tumor growth and metastasis [5]. Based on these observations it has been proposed that during tumorigenesis, BAMBI upregulation allows tumor cells to escape growth arrest by TGFβ, and to activate growth and increase tumor cell motility and invasion [6].

As mRNA for BAMBI is co-expressed with bone morphogenic proteins BMP, TGFβ, and activin during development, it was thought to play an important role in development [7]–[9]. Furthermore the Wnt pathway is also essential for development [10]. It was therefore quite unexpected when mice with germ-line elimination of the BAMBI gene were born without detectable developmental abnormalities and reproduced normally [11]. Functional analysis of the BAMBI-deleted (*BAMBI^−/−^*) mice have not been reported. Surprisingly in zebrafish a role for BAMBI was identified in platelet-endothelial interaction and thrombus formation after endothelial injury [12].

In mice the significance of BAMBI's interaction with TGFβ signaling and the resulting effects on fibrosis were recently examined in models of progressive liver fibrosis. In these experiments a novel link was proposed connecting the innate immune system via TLR4 activation directly to TGFβ signaling without intermediate changes in cytokine or chemokine induction or inflammatory cell involvement. TLR4 activation by LPS endotoxin downregulated BAMBI specifically in hepatic stellate cells. The downregulation of BAMBI was shown both at the mRNA and protein level, and sensitized the cells to TGFβ, i.e. it enhanced the profibrotic effects of TGFβ. These effects could not only be demonstrated in vitro in stellate cells, but also in vivo in models of liver cirrhosis.

These data are exciting as TGFβ is considered the single most important cytokine in progressive organ fibrosis, including in the kidney. Progressive kidney disease may affect as much as 25% of the aging population in the US and also represents an independent major cardiovascular risk factor. Therefore research on potential ways of modifying the detrimental influence of TGFβ is of major interest in kidney disease. Apart from TGFβ, Wnt signaling molecules also act as potent regulators for renal tissue morphogenesis and pathogenesis in renal injury [13]. Wnt signaling is important for angiogenesis and survival of endothelial and mesangial cells [14], [15]. As BAMBI may influence both TGFβ and Wnt signaling, we thought to obtain information on BAMBI's distribution and regulation within the kidney. At present the only published information on BAMBI and the kidney is restricted to the presence of mRNA for BAMBI in whole rat kidney [16]. Surprisingly there also have been no reports on the localization of BAMBI within organs, a prerequisite to examine its cell type-specific expression and function. This is all the more important as the effects of both TGFβ and Wnt are exquisitely cell type-dependent and as BAMBI would have to be present in the same cell type. We therefore set out to characterize the expression of BAMBI mRNA and protein in adult murine and human kidney and to localize it to specific kidney structures and cells. Much to our surprise we found BAMBI expression restricted to vascular compartments, such as the glomerulus and renal blood vessels, and therein specifically to endothelial cells. Furthermore in cultured murine microvascular glomerular endothelial cells and in HUVECs, endotoxin did not down-modulate BAMBI mRNA as would be expected from the data reported in liver stellate cells [2]. Also unexpectedly for a putative transmembrane protein, BAMBI mRNA had a short half life of about 60 minutes and was regulated by post-transcriptional mechanisms inhibitable by cycloheximide. In experiments looking at the regulation of BAMBI protein turnover in endothelial cells, we found predominant lysosomal and autolysosomal, but not proteasomal pathways involved in BAMBI degradation. Thus in the kidney BAMBI's role may be restricted to endothelial function, a novel and potentially important finding, as the endothelium is also a major target for TGFβ and Wnt effects in health and disease.

## Methods

### Ethics Statement

For the human kidney immunohistology, informed written consent was obtained from all patients allowing the use of material left over from diagnostic purposes to be used for research. The experimental protocol was approved by the local internal review board, the Ethic Committee of Fondazione IRCCS Ca'Granda Ospedale Maggiore Policlinico. For the characterization of human glomeruli and tubules, renal biopsies were obtained after written consent and approval of the ethics committee and in the frame of an international multicenter study, the European Renal cDNA Bank-Kroener-Fresenius biopsy bank, approved by the specialized subcommittee for internal medicine of the Cantonal Ethics Committee of Zurich.

### Mice

BAMBI heterozygote (*BAMBI* ^+/−^) male and female mice were generated by Rulang Jiang as published [11] and were kindly provided by Robert Schwabe (Columbia University). The mice are in C56BL/6;129svj background and were intercrossed to obtain wild-type (*BAMBI* ^+/+^) and BAMBI-deleted (*BAMBI* ^−/−^) mice. Wild type C57BL/6 mice were obtained from Taconic Farms. Generation of these mice and genotyping protocols have been described previously [11]. All animal work was approved under the protocol LA08-00399: Modulators of Renal Fibrosis by the IACUC, the Institutional Animal Care and Use Committee that ensures that all vertebrate animal studies performed at MSSM are conducted in accordance with Animal Welfare Act regulations and Public Health Service policies, and that such studies conform to the Institution's Assurance1 document filed with the Office of Laboratory Animal Welfare of the National Institutes of Health.

### Cell culture

293T cells and HUVECs were purchased from ATCC and cultured in DMEM with 10% FBS and 1% Penicillin-Streptomycin. A murine mesangial cell line was cultured in DMEM with 5% FBS and 1% Penicillin Streptomycin at 37°C as previously reported [17]. The murine glomerular endothelial cells generated and characterized by Akis and Madaio [18] were cultured in RPMI with 10% FBS and 1% Penicillin-Streptomycin under permissive conditions at 33°C in the presence of 100U/mL IFN-γ (Cell Sciences). For differentiation cells were incubated at 37°C without INF-γ. Murine renal tubular epithelial cells and murine podocytes were cultured as previously reported [19].

### Reagents

The following reagents were used: cycloheximide (1mg/ml, Sigma), endotoxin LPS (1ug/ml, Sigma; from *Escherichi Coli* serotype 026:B6), bafilomycin A1 (1µM) from *Streptomyces griseus*, Sigma), 3-methyladenine (10mM, Sigma), MG132 (50µM, Cayman Chemical), epoxomicin (10µM, Sigma), and rapamycin (10µg/ml, Sigma). For the experiments the respective controls received the same final concentration of the vehicle used for the experimental agent.

### Antibodies

The primary antibodies used in these studies were as follows: goat polyclonal anti-mouse BAMBI (R&D Systems), rat monoclonal anti-mouse CD31 (Santa Cruz Biotechnology), mouse monoclonal anti-human BAMBI (Abnova), mouse monoclonal anti-rat synaptopodin (Progen, Heidelberg, Germany), rabbit polyclonal anti-human synaptopodin (Sigma-Aldrich), rabbit polyclonal anti-human EEA1 (Cell Signaling Technology), mouse monoclonal anti-bovine ubiquitin (Zymed laboratories), rabbit polyclonal anti-human LC3 (Novus Biologicals), and mouse monoclonal anti-rabbit GAPDH (Santa Cruz Biotechnology). The secondary antibodies used in these studies were as follows: anti-goat AlexaFluor 488 (Invitrogen), anti-goat AlexaFluor 568 (Invitrogen), anti-rat FITC (Abcam), and donkey-anti-goat, chicken-anti-goat, and goat-anti-mouse horseradish peroxidase-conjugated antibodies (R&D Systems).

### Plasmids

Murine BAMBI in pAdTrack CMV vector [2] were kindly provided by Robert Schwabe (Columbia University) and subcloned into the BamH1 site of the HIV1-lentiviral vector [20]. Xenopus BAMBI in pCS2^+^ vector was a gift from Christoph Niehrs, DKFZ, Heidelberg, Germany.

### Transient Transfection of HEK 293T cells

Cells were plated in 6-well dishes and transfected either with murine full-length BAMBI or xenopus full-length BAMBI using Effectene (Qiagen) following the manufacturers protocol. 48 hours following transfection cells were lysed with RIPA buffer on ice (1M Tris pH7.4; 5M NaCl; 0.5M EDTA pH 7.4; 10%SDS containing protease inhibitors). Lysates were stored at −20°C.

### Lentiviral system for HUVECs expressing murine BAMBI

To package the virus, HEK293T cells were transfected with a lentiviral vector carrying the murine BAMBI gene with a FLAG-tag and a pCMVG plasmid containing the viral envelope gene together with a plasmid containing gag/pol using Lipofectamine 2000 reagent (Invitrogen). Viral particles were harvested after filtering through a 0.45µM filter and used to infect HUVECs to generate a cell line expressing BAMBI. Infected cells were monitored by expression of IRES-GFP from the bicistronic lentiviral vector that was under the control of an internal spleen focus-forming virus (SFFV) promoter and transfection efficiency was determined to be in the range of 80–90%.

### Immunostaining

Mice kidneys were embedded in OCT compound (Tissue-Tek, Andwin Scientific) directly after nephrectomy, snap-frozen in isopentane (Fisher) over liquid nitrogen and stored at −80°C. Frozen sections (4µm) were post-fixed in cold acetone (Fisher), washed with PBS, and blocked with a solution of 2% BSA (Fisher) and 0.2% Fish Gelatin (GE Healthcare) in PBS. Primary double staining was performed with goat-anti-BAMBI (10µg/mL) and rat-anti-CD31 (1µg/mL) overnight. Slides were washed with PBS and incubated with secondary antibodies AlexaFluor 568 and FITC for one hour. Sections were subsequently treated with DAPI for nuclear counter stain. The sections were mounted on slides using Fluoromount-G. Fluorescent images were acquired using the Zeiss Axioplan2 fluorescence microscope.

Cultured HUVECs expressing BAMBI were grown on Lab-Tek 8-well Permanox® slides (Nunc), washed with PBS, fixed in 4% paraformaldehyde (Sigma) and permeabilized with 1% Triton X-100 (Bio-Rad). Cells were stained with goat-anti-BAMBI (2µg/mL) and rabbit-anti-LC3 (10µg/mL) antibodies followed by incubation with AlexaFluor 568 and AlexaFluor 488 secondary antibodies. Fluorescent images were acquired using the Zeiss Axioplan2 fluorescence microscope and the Leica SP5 DMI confocal microscope.

For the human kidney immunohistology the unfixed healthy renal tissue from a tumor nephrectomy was embedded in OCT, snap-frozen in a mixture of isopentane and dry ice and stored at −80°C. Cryosections (5um) were fixed in cold acetone, rinsed, and sequentially incubated with the primary antibody mouse-anti-BAMBI followed by the proper fluorescent-tagged secondary antibody (AlexaFluor 488 or AlexaFluor 546). For double stainings, sections were first incubated with the first primary antibody (mouse-anti-BAMBI and AlexaFluor 546); after adequate washing, the procedure was repeated with the second primary antibody (rabbit-anti-synaptopodin and AlexaFluor goat anti-rabbit IgG). Sections were mounted with anti-fading Mounting Medium (Fluorsave; Calbiochem, VWR International). Specificity of labeling was demonstrated by the lack of staining after substituting phosphate-buffered saline and proper control IgGs (rabbit primary antibody isotype control and mouse primary antibody isotype control, both from Invitrogen) for the primary antibody. Tissue from mice with genetic deletion of the BAMBI gene served as additional controls.

### Immunoblotting

Kidney tissue and cultured cells were lysed in ice-cold lysis buffer (1M Tris pH7.4; 5M NaCl; 0.5M EDTA pH 7.4; 10%SDS containing Complete Mini protease inhibitor cocktail (Roche)) according to the manufacturer's instructions, and the proteins were separated on a 12% SDS-PAGE gel as described previously and transferred onto PolyScreen® PVDF Hybridization Transfer Membrane (Perkin-Elmer) using a semi-dry transfer cell. After transfer, membranes were incubated with one of the following primary antibodies: goat-anti-BAMBI (0.8 µg/mL), mouse monoclonal LC3 (2 µg/mL), or mouse monoclonal GAPDH (0.1 µg/mL), followed by incubation with respective horseradish peroxidase conjugated secondary antibodies: chicken-a-goat and goat-a-mouse. Membranes were developed using normal- and high-sensitivity versions of the Supersignal West Pico Chemiluminescent substrate (Thermo Scientific). For quantification, band intensity was measured using the NIH ImageJ program.

### Induction of autophagy and immonufluorescence staining

To induce autophagy, cells grown on Lab-Tek chamber slides were either serum-starved or treated with rapamycin for 24 hours. Inhibitors to autolysosomal proteases or autophagy were used. Fixed and stained cells were imaged as described above. Using Metamorph software, quantification of colocalization of BAMBI and LC3 was done by outlining each cell and measuring the areas of overlap for green and red, and results were expressed as percentage overlap. For each measurement 6–8 cells per treatment were quantified and averaged.

### *In situ* hybridization

Tissues were deparaffinized and rehydrated, then permeabilized with saponin-EBSS [21]. Postfixation was carried out with 4% buffered paraformaldehyde. Sections were then acetylated with a solution of acetic-anhydride/triethanolamine. After washing, a prehybridization solution, containing 10× standard sodium citrate (SSC), 10× Denhardt's solution, 50% formamide, 10% Dextran sulfate and salmon sperm DNA (200 ug/mL, all from Sigma), was applied for one hour. Sense and antisense biotin-labeled oligonucleotides (IDT, Tema Ricerca SrL, Bologna, Italy) specific for BAMBI human sequence (sense: GAT GTC TGT CGT GCT TGC AAG AGA GTC CAG GCA GCC ATG GGT GAG TGG GGA ATT TGA GTT CTG AGG ATC AAG AAG TCT AGA GAA GCA GGC GCT GAG CTCA; antisense: TGA GCT CAG C GC CTG CTT CTC TAG ACT TCT TGA TCC TCA GAA CTC AAA TTC CCC ACT CAC CCA TGG CTG CCT GGA CTC TCT TGC AAG CAC GAC AGA CAT C) were appropriately diluted in the prehybridization solution and applied overnight in a water-saturated atmosphere. After hybridization, stringency washes were done with 4× SSC, 2× SSC–50% formamide at 45°C, and again with 1× SSC at room temperature for 30 minutes. After incubation in peroxidase quenching solution (3% H_2_O_2_), specimens were incubated with alkaline phosphatase-labeled streptavidin, and the enzymatic reaction was developed with Fast Red (Sigma-Aldrich). After washing in distilled water, sections were mounted with glycerol (Fisher).

### Isolation of Glomeruli

Human renal biopsy specimens were procured in an international multicenter study, the European Renal cDNA Bank-Kroener-Fresenius biopsy bank. Following renal biopsy, the tissue was transferred to RNase inhibitor and microdissected into glomerular and tubular fragments. Total RNA was isolated from microdissected glomerular and tubulointerstitial tissue and underwent reverse transcription according to a protocol previously reported [22]. Murine glomeruli and tubular preparations were isolated from mice using magnetic separation as previously described [23]. The isolated glomeruli were then used for mRNA analysis (see below) and protein analysis by homogenizing them in cell lysis buffer.

### Quantitative Real-Time PCR analysis

Total RNA from kidney, glomeruli, tubuli and cultured cells were isolated using Qiagen RNeasy mini columns. The cDNA templates were synthesized using the SuperScript II Reverse Transcriptase First-Strand cDNA Synthesis kit (Invitrogen) and subsequently amplified via quantitative polymerase chain reaction (qPCR) using SYBR Green PCR Master Mix (Applied Biosystems). qPCR reactions were performed with the ABI Prism 7900HT Fast Real-Time PCR System and Sequence Detection Systems Enterprise Database with 384 wells (Applied Biosystems Inc) with an absolute quantification template provided by the manufacturer, using the primers in Table 1, with GAPDH, Actin, and 18S as housekeeping genes to normalize the sample amount. Fold change in gene expression was determined using the 2^−ΔΔC^_T_ method as previously described [24]. Actual transcript copy number was determined during the qPCR assay using the Applied Biosystems Sequence Detection Software and a standard dilution curve to establish a linear relationship between CT value and copy number of each reaction. Copy number was normalized to copy number of housekeeping genes. Additionally, qPCR was performed on some samples by the TaqMan method as reported earlier [22]. Pre-developed TaqMan reagents were used for human BAMBI, podocin, as well as the reference gene GAPDH (Applied Biosystems). The mRNA expression was analyzed by standard curve quantification.

**Table 1:** Primer pairs used for quantitative RT-PCR.

GENE	FORWARD	REVERSE
mACTIN	5′-ACCGTGAAAAGATGACCCAG-3′	5′-AGCCTGGATGGCTACGTACA-3′
mGAPDH	5′-AACTTTGGCATTGTGGAAGG-3′	5′-ACACATTGGGGGTAGGAACA-3′
18S	5′-AAGGAGACTCTGGCATGCTAAC-3′	5′-CAGACATCTAAGGGCATCACAGAC-3′
mBAMBI	5′-AACAGGCCCAAAACCACTCTG-3′	5′-TTGTCCTGAGGCTTCGCTCTT-3′
mTLR2	5′-CACCGGTCAGAAAACAACTTACC-3′	5′-CAAGATCCAGAAGAGCCAAAGAG-3′
mTLR4	5′-TTCAGAACTTCAGTGGCTGGATT-3′	5′-CCATGCCTTGTCTTCAATTGTTT-3′
mTGFBRI	5′-AAATTGCTCGACGCTGTTCT-3′	5′-CAACCGATGGATCAGAAGGT-3′
mTGFBRII	5′-GCATCCAGATCGTGTGTGAG-3′	5′-ACTGTGCTGTTAGACGGGCT-3′
hACTIN	5′-ACCGCGAGAAGATGACCCAG-3′	5′-AGCCTGGATAGCAACGTACA-3′
hBAMBI	5′-TGCACGATGTTCTCTCTCCT-3′	5′-GAAGTCAGCTCCTGCACCTT-3′
hTLR2	5′-CTACTGGGTGGAGAACCTTATGGT-3′	5′-CCGCTTATGAAGACACAACTTGA-3′
hTLR4	5′-GGCATGCCTGTGCTGAGTT-3′	5′-CTGCTACAACAGATACTACAAGCACACT-3′
hTGFBRI	5′-TGTTGGTACCCAAGGAAAGC-3′	5′-TGCCAGTCCTAAGTCTGCAAT-3′
hTGFBRII	5′-GCCTGGTGAGACTTTCTTCA-3′	5′-GAGGCTGATGCCTGTCACTT-3′

### Statistical analysis

Values are expressed as means ± SEM, and significance established by student's *t* test. Statistical significance was defined as p<0.05.

## Results

### BAMBI mRNA and protein is expressed in endothelial cells in the kidney

**Figure 1:**
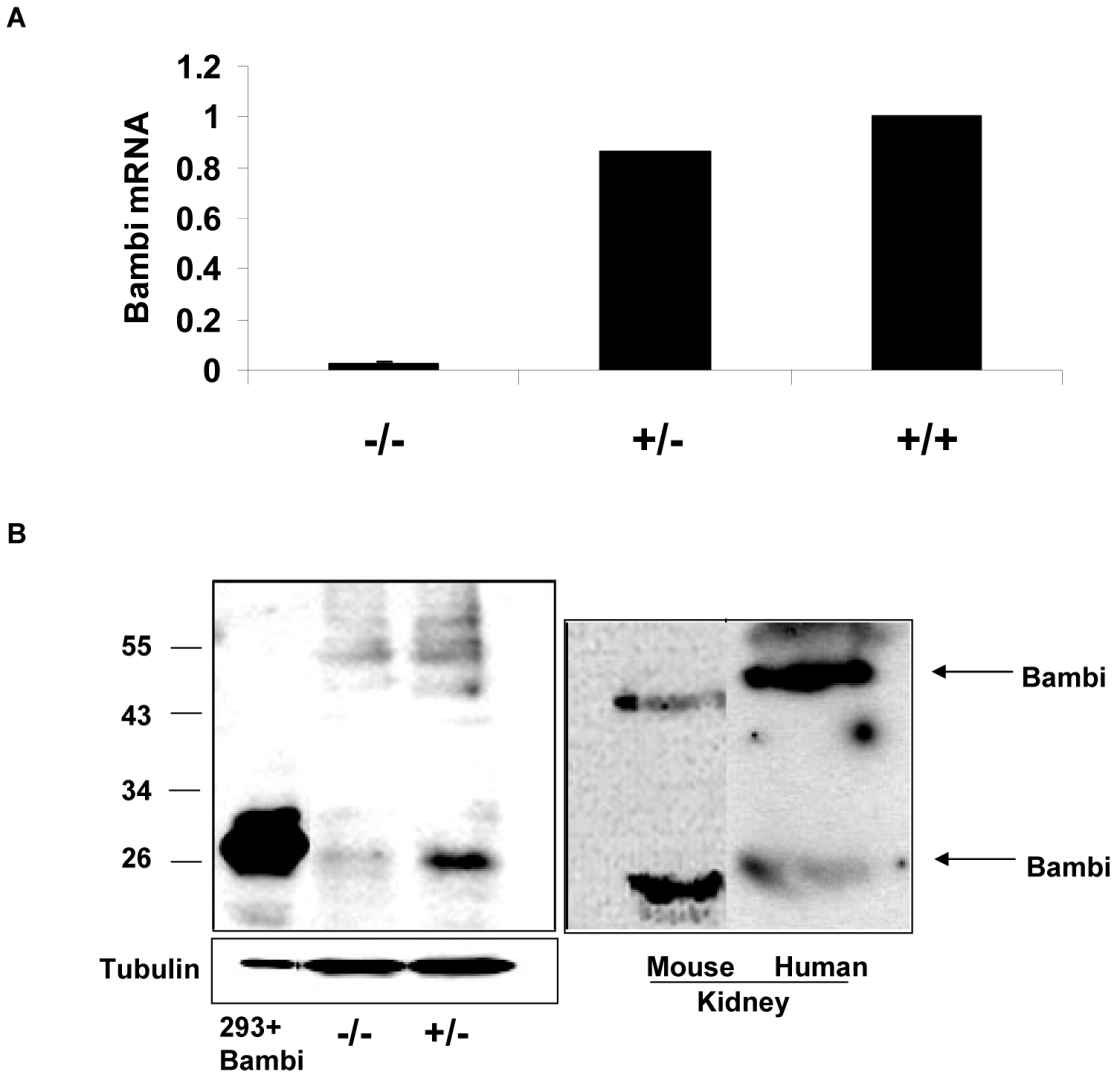
Renal Levels of mRNA for BAMBI. **1A**. Comparison of relative expression for BAMBI mRNA in kidneys from wild type (*BAMBI^+/+^*), heterozygous (*BAMBI^+/−^*), or BAMBI-deleted (*BAMBI^−/−^*) mice expressed as fold change relative to *BAMBI^+/+^* controls. **1B**. Western blot for BAMBI protein in kidney lysates from *BAMBI^+/−^*, *BAMBI^−/−^*, as well as *BAMBI^+/+^* mice and normal human kidney. For localization of BAMBI, lane 1 shows lysate from HEK 293 cells overexpressing BAMBI. Please note that the human BAMBI travels at a molecular weight around 28–29 kDa as compared to the mouse BAMBI with a molecular weight of 26–27 kDa. Tubulin mRNA is shown as a loading control for the *BAMBI^+/−^* and *BAMBI^−/−^* kidney lysates.

BAMBI can modulate TGFβ action, and TGFβ action is very cell-specific, including as the major modulator of progression of renal diseases [25]. The cell-specific expression of BAMBI is therefore of considerable interest. So far BAMBI mRNA levels have been described for whole organs, but no data on cell-specific expression of BAMBI in any organ, including kidney, are available. Therefore we examined as a first step the intrarenal expression of BAMBI both in murine and human kidney. Expression of mRNA for BAMBI could be demonstrated in whole mouse kidney extracts (Figure 1). No mRNA for BAMBI was detected in kidney from *BAMBI^−/−^* mice (Figure 1A). The mRNA data were corroborated by protein levels for BAMBI in Western blots with BAMBI overexpressed in HEK293 cells serving as control for the specificity and position of the BAMBI protein (Figure 1B). Consistent with the mRNA data, BAMBI protein could be visualized by Western blot in extracts from both mouse and human kidney. Besides the band colocalizing with the 26–28 kDa size of the overexpression BAMBI standard, varying amounts of a band with about 54 kDa were noted. This band of about 54 kDa may represent a dimer resistant to SDS and reducing conditions, as originally apparent in the paper reporting on the cloning of BAMBI [1]. The specificity of 26–28 and 54 kDa bands reacting with the antibody against BAMBI is supported by their absence in the kidney extracts from the *BAMBI^−/−^* mice (Figure 1B).

**Figure 2:**
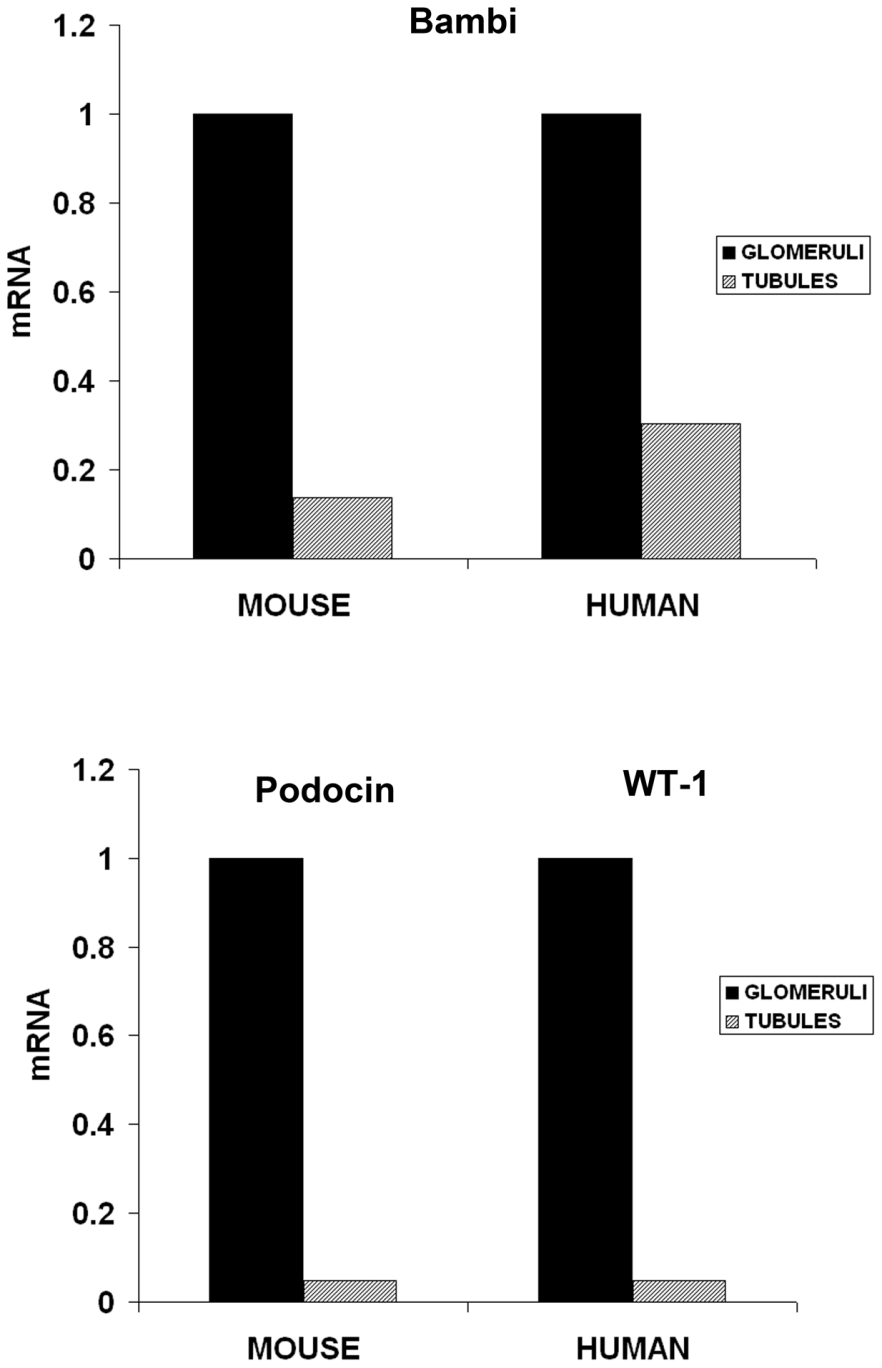
Levels for mRNA of BAMBI in tubules and glomeruli. Comparison of relative mRNA levels for BAMBI in isolated glomerular and tubular fractions from murine and human kidneys. Levels of glomerular-specific podocyte marker genes, podocin or WT-1, respectively, indicate their enrichment in the glomerular preparation and their low levels in the tubular preparations. Values are means +/− SEM of 3–4 experiments for each set.

**Figure 3:**
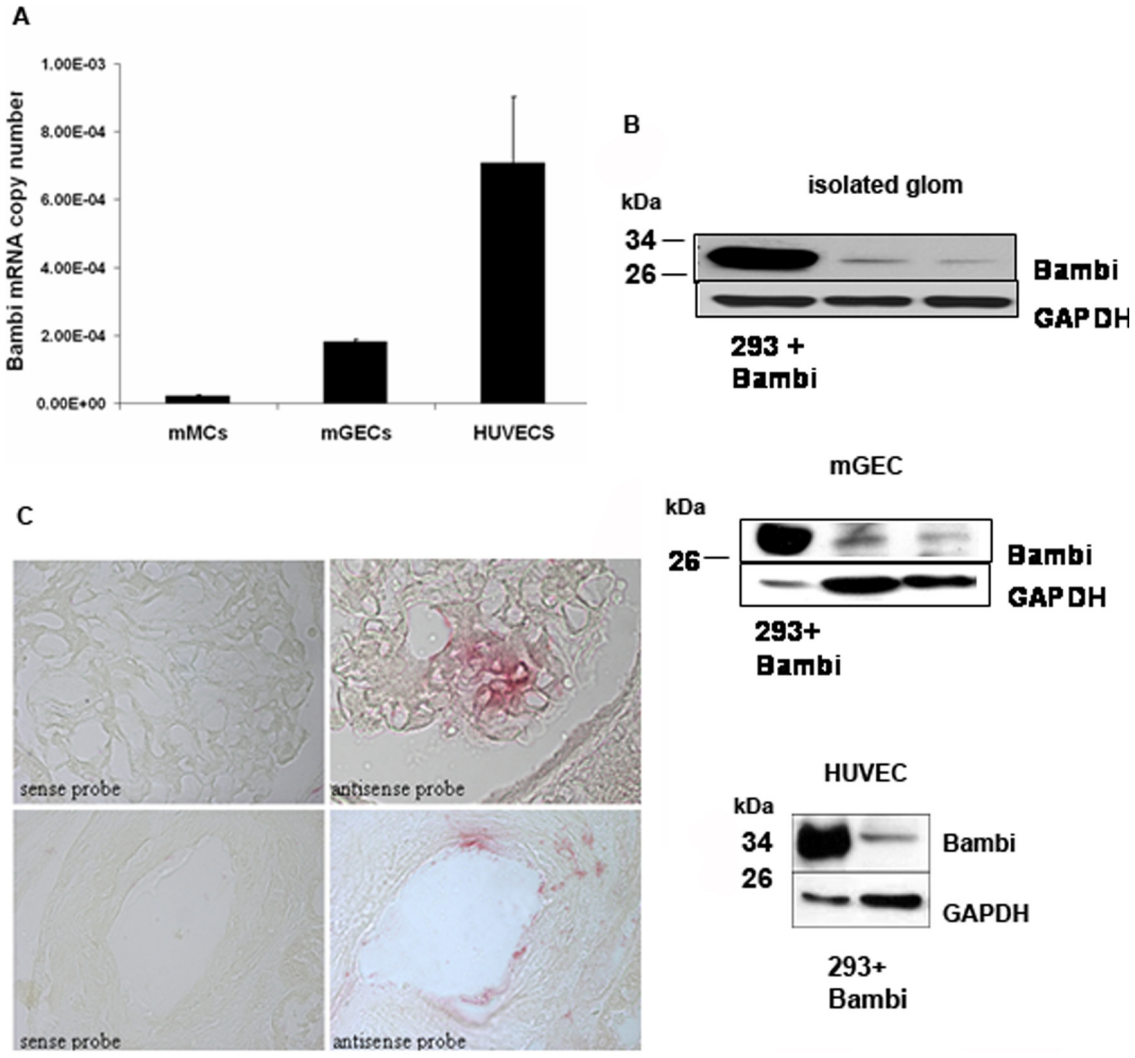
Levels of mRNA for BAMBI in glomerular cell types. **3A**. BAMBI mRNA levels as copy numbers (means +/− SEM) in murine mesangial cells (mMC; n = 4), murine glomerular endothelial cells (mGEC; n = 3) or human umbilical vein endothelial cells (HUVEC; n = 14). **3B**. Western blot for BAMBI in glomeruli isolated from mouse kidney, in cultured mGECs and HUVECs. For comparison the position of murine BAMBI overexpressed in HEK 293 cells is shown. Blot for GAPDH is shown as loading control. **3C**. In situ hybridization for BAMBI in human renal blood vessel and in human glomerulus. Positive signals are only obtained with the antisense, but not with the sense probe and localize to endothelial cells in the blood vessel and in glomerular capillary loops.

To analyze BAMBI's distribution within the kidney, the major intrarenal structures, i.e. tubules and glomeruli, were isolated from mouse and human kidney and analyzed by real-time RT-PCR. As shown in Figure 2 BAMBI mRNA was predominantly expressed in isolated glomeruli and much less in the tubular compartment of both mouse and human kidneys. The purity of the murine glomerular and tubular isolates was determined by expression of aquaporin as a marker for tubular epithelium [26] (results not shown) and WT-1 or podocin as markers of podocytes [27] a cell type restricted to glomeruli ([19]; Figure 2). Glomeruli contain three different cell types: podocytes as visceral epithelial cells, mesangial cells as modified smooth muscle cells or pericytes, and endothelial cells [28]. We therefore examined these cell types by using well-characterized murine cell lines of podocytes [29], mesangial cells [17], and glomerular endothelial cells [18]. For the tubular cells we used a murine proximal tubular epithelial cell line [30], [31]. By real-time RT-PCR, the expression of BAMBI in podocyte and tubular epithelial cell lines was too low to allow reliable determination. Levels of mRNA for BAMBI were consistently detectable at a low level in murine mesangial cells, but were markedly higher in differentiated glomerular endothelial cells (mGEC; Figure 3A). Because of the BAMBI expression in murine microvascular endothelial cells mGEC, we also examined a cell line derived from human umbilical cord vascular endothelial cells, HUVECs [32]. HUVECs also had considerable levels of mRNA for BAMBI, consistent with BAMBI expression specifically by endothelial cells (Figure 3A). Both the HUVECs and mGECs used were positive for CD31 as a marker of endothelial cells (results not shown). Again protein determination by Western blot supported the mRNA results. The level of BAMBI protein in podocytes and tubular epithelial cells was undetectable, confirming the absence of mRNA for BAMBI in these cell types. Also in cultured mMC, BAMBI protein levels were too low for consistent and reliable detection by Western blot. A specific band for BAMBI could, however, be demonstrated in isolated mouse glomeruli, and both in mGEC and HUVECs (Figure 3B), consistent with the robust expression of mRNA for BAMBI in these cells (Figure 3A).

### Localization of BAMBI by immunohistology in murine and human kidney and by *in situ* hybridization in human kidney

**Figure 4:**
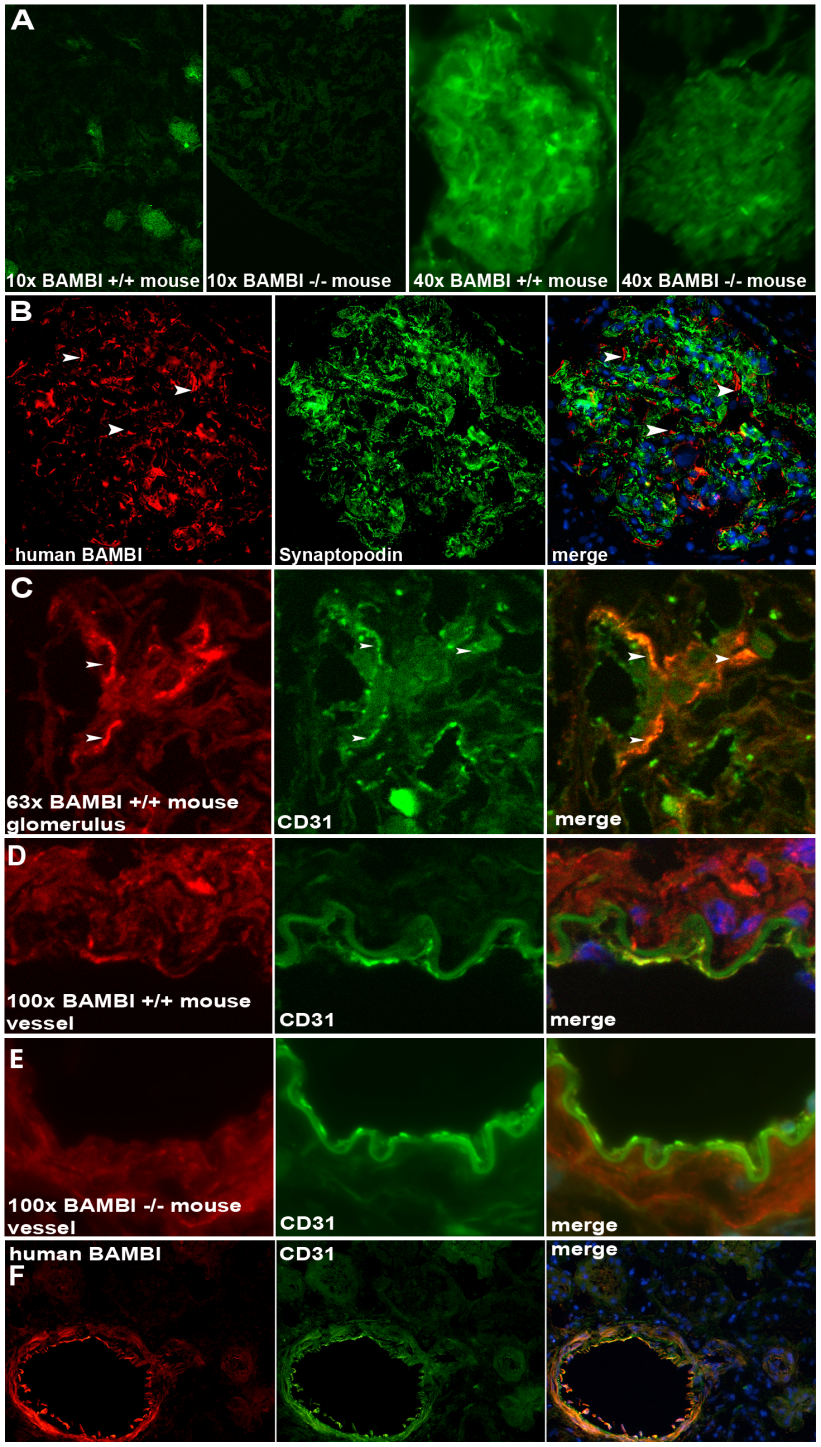
Immunohistological localization of BAMBI in kidney. **4A**. Immunofluorescence staining for BAMBI in frozen sections from kidneys of *BAMBI^+/+^* and *BAMBI^−/−^* mice (10× and 40× magnification). At low power, glomeruli are positive for BAMBI staining (green) in the *BAMBI^+/+^* kidneys, while only background fluorescence is seen in kidney from *BAMBI^−/−^* mice. At high magnification the staining for BAMBI is seen in a glomerulus of the *BAMBI^+/+^* kidney, whereas only background fluorescence is apparent in the glomerulus from *BAMBI^−/−^* mice. **4B**. Immunofluorescence staining for BAMBI (red) and synaptopodin (green) as a podocyte-specific marker in frozen sections from human kidney with an endothelial distribution and lack of colocalization (arrows) with synaptopodin. **4C**. Double immunofluorescence staining for BAMBI (red) and CD31 (green) as an endothelial marker and the merged picture in a glomerulus at high magnification from a frozen section from a kidney of a *BAMBI^+/+^* mouse. The specific co-staining for BAMBI and CD31 is apparent in the merged picture as yellow/orange on the luminal aspects of the glomerular capillaries, as indicated by arrows. **4D**. Double immunofluorescence staining for BAMBI (red) and CD31 (green) and the merged picture in a renal medium-sized artery at high magnification from a frozen section from a kidney of a *BAMBI^+/+^* mouse The specific co-staining for BAMBI and CD31 is apparent in the merged picture as yellow/orange on the luminal aspects of the vascular endothelium. **4E**. Double immunofluorescence staining of a section of an artery from a *BAMBI^−/−^* mouse. Only some faint, non-specific, red background staining is apparent for BAMBI in the *BAMBI^−/−^* section, whereas endothelial CD31 staining remains strong. **4F**. Double immunofluorescence staining in human kidney showing positive staining for BAMBI and CD31 in the endothelium of a mid-sized artery with colocalization as shown in the merged picture (yellow/orange).

For immunohistological localization of BAMBI we used frozen sections from kidneys of littermate *BAMBI^+/+^*, *BAMBI^+/−^*, and *BAMBI^−/−^* mice. In wild type kidneys BAMBI staining was predominantly in the glomeruli (Figure 4A), consistent with the mRNA and Western blot data. In kidneys of *BAMBI^−/−^* mice only background staining was noted, comparable to what was seen with omission of the BAMBI antibody and use of only the secondary antibody. The glomerular staining pattern was consistent with an endothelial distribution. Besides in glomeruli, there were also small spots of BAMBI staining in a peritubular location, that may reflect peritubular capillaries. A comparable pattern of staining was observed in frozen sections of normal human kidney tissue. BAMBI was present in glomeruli with an endothelial pattern and without any overlap with synaptopodin staining specific for podocytes (Figure 4B). In order to further confirm the endothelial localization, we performed double staining for BAMBI and CD31 as an endothelial marker in glomeruli from *BAMBI^+/+^* mice. CD31 stained the endothelium of glomerular capillaries, as shown at high magnification in Figure 4C. Upon merging of the CD31 and the BAMBI immunofluorescence, the signals colocalized at the luminal aspects of glomerular capillaries, consistent with endothelial localization of BAMBI (Figure 4C). Staining of CD31-positive peritubular capillaries for BAMBI was variable and faint, with only about 10–20% being double-positive. This may either represent differences in BAMBI expression in different microvascular endothelia, or may be due to low intensity of staining in peritubular capillaries. The endothelial localization of BAMBI could be clearly shown by double staining for BAMBI and CD31 in mid-sized arteries and veins from mouse or human kidneys (Figure 4D and F). As to be expected, BAMBI staining was absent in the endothelium of blood vessels from *BAMBI^−/−^* kidneys, while CD31 staining remained positive (Figure 4E). Taken together these immunohistological data show a specific staining for BAMBI in glomerular endothelial cells and in the endothelial cells of blood vessels of both mouse and human kidney.

To further confirm the localization of BAMBI to endothelial cells in the human kidney, we performed in situ hybridization for mRNA of BAMBI. As shown in Figure 3C a specific signal for mRNA of BAMBI was observed only in endothelial cells of blood vessels and of glomeruli, where it could be shown in some, but not all capillary loops (Figure 3C). This is to be expected as endothelial nuclei will only be prominently positioned occasionally on a glomerular section. Taken together the in situ hybridization data confirm the restricted expression of BAMBI in endothelial cells noted by immunohistology and by the mRNA data with cultured endothelial cells.

### Regulation of mRNA for BAMBI, TβRI, TβRII, TLR2 and TLR4 in mMC, mGEC, and HUVEC

**Figure 5:**
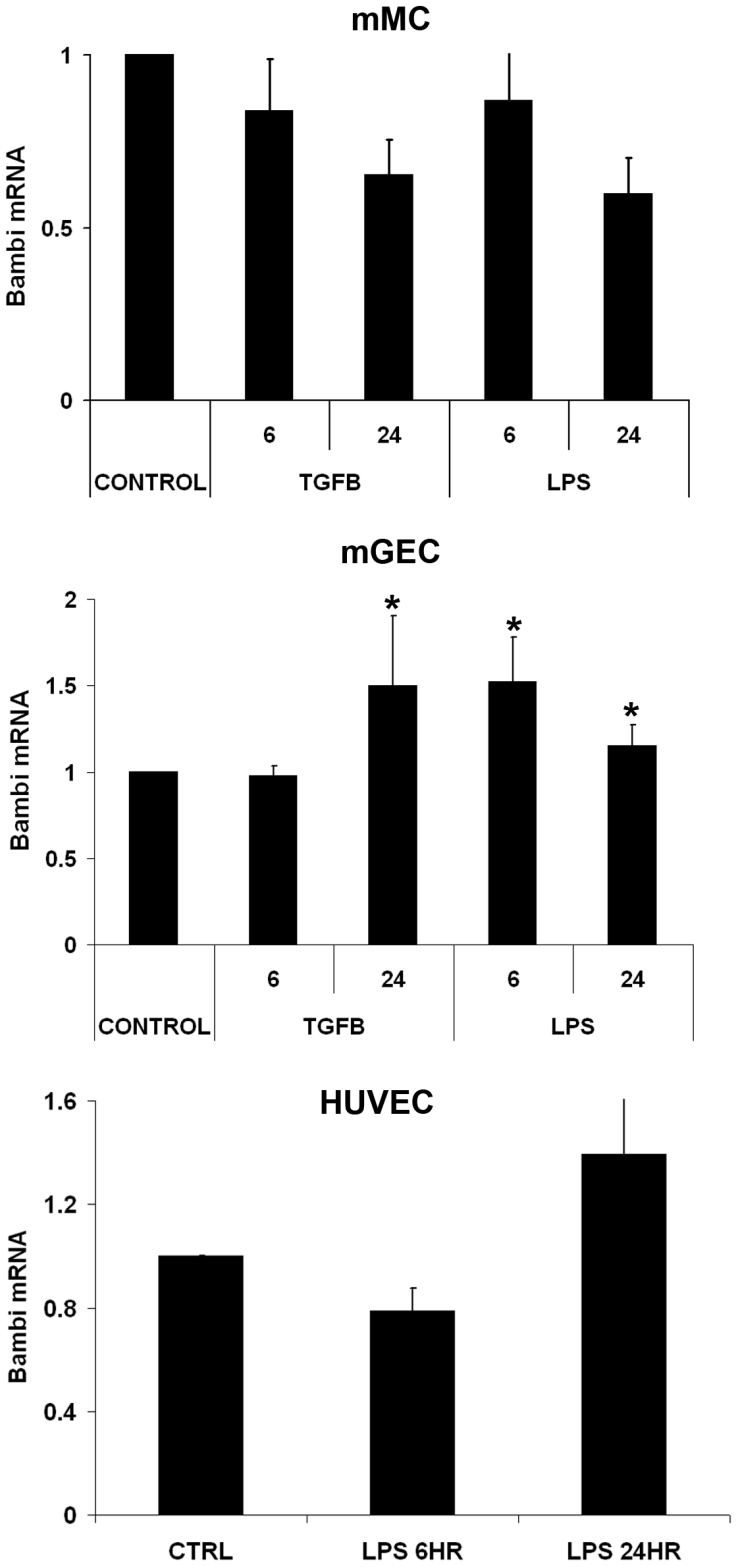
Effects of TGFβ and LPS on BAMBI transcription. Effects of TGFβ or LPS on the mRNA levels for BAMBI in murine mesangial cells (mMC) and murine glomerular endothelial cells (mGEC) and human umbilical vein endothelial cells (HUVEC). Cells were incubated under control conditions or with the addition of TGFβ (5 ng/ml) or LPS (1 µg/ml) for 6 and 24 hours. Data for mRNA are corrected for actin expression and calculated as fold change compared to the respective untreated controls. Values represent means +/− SEM from 3–7 independent series of experiments with TGFβ and of 3–13 series with LPS. Asterisks indicate p<0.05 as compared to the respective controls.

In HepG2 hepatocellular carcinoma cells, TGFβ has been reported to increase mRNA levels for BAMBI [33], whereas endotoxin (LPS) downregulated mRNA for BAMBI in murine hepatic stellate cells via TLR4 [2]. We therefore examined the effects of TGFβ and of LPS on the mRNA levels of BAMBI. In parallel we determined mRNA levels of TLR2 and 4 as receptors for LPS, and of the TGFβ receptors TβRI and II in mMC and mGEC. As shown in Figure 5 in mMCs, TGFβ had no effect on mRNA for BAMBI. LPS (1ug/ml) also failed to consistently alter mRNA levels for BAMBI in mMC after 6, 12, and 24 hours of incubation (Figure 5). Dose response experiments with LPS from 0.01 to 10ug/ml also showed no effects on BAMBI mRNA levels (results not shown). LPS also did not change mRNA for TβRI or TβRII or TLR4 after 6 and 24 hours, but slightly reduced mRNA for TLR2 (Table 2). We next examined BAMBI in differentiated mGECs. TGFβ caused a slight increase (1.4 fold; p<0.005) in BAMBI mRNA levels after 24 hours of incubation. TGFβ increased TβRI mRNA after 6 hours, but no longer at 24 hours of incubation (Table 2). TGFβ robustly downregulated TβRII after 6 and 24 hours of incubation (Table 2). TGFβ upregulated TLR2 at 6 hours, but had no consistent effect on TLR4 either at 6 or 24 hours (Table 2). LPS significantly (p<0.05) increased levels of mRNA for BAMBI in mGECs after 6 and 12 hours, but no longer after 24 hours of incubation (Figure 5). LPS significantly increased mRNA levels for TβRI at 12 hours without significantly changing those for TβRII (Table 2). LPS increased mRNA for TLR2 and to a lesser extent those for TLR4 as well (Table 2). In HUVEC, incubation with LPS (1 µg/ml) did not significantly alter BAMBI expression after 6 and 24 hrs of incubation (Figure 5).

**Table 2:** Fold change values for BAMBI mRNA in response to TGFβ and LPS.

*mMC*					
GENE	CTRL	TGFβ		LPS	
*(fold change)*		*6 hrs*	*24 hrs*	*6 hrs*	*24 hrs*
**TBRI**					
*mean ± sem*	1±0	1.68±0.33**	1.87±0.59**	1.23±0.17	1.09±0.36
*n*		7	4	15	4
**TBRII**					
*mean ± sem*	1±0	0.83±0.17	0.66±0.21	1.21±0.17	0.86±0.21
*n*		10	4	18	5
**TLR-2**					
*mean ± sem*	1±0	1.16±0.26	0.76±0.17	0.73±0.15	0.72±0.08
*n*		7	4	9	5
**TLR-4**					
*mean ± sem*	1±0	0.58±0.13***	0.64±0.12***	1.01±0.15	0.94±0.23
*n*		7	4	15	5

*mGEC*					
GENE	CTRL	TGFβ		LPS	
*(fold change)*		*6 hrs*	*24 hrs*	*6 hrs*	*24 hrs*
**TBRI**					
*mean ± sem*	1±0	1.85±0.45*	1.21±0.12	1.27±0.19	1.46±0.42***
*n*		3	3	8	3
**TBRII**					
*mean ± sem*	1±0	0.38±0.03***	0.41±0.08***	1.49±0.39	1.08±0.01
*n*		3	3	8	3
**TLR-2**					
*mean ± sem*	1±0	3.66±0.63***	1.42±0.06	2.18±0.59*	2.02±0.66*
*n*		3	3	4	3
**TLR-4**					
*mean ± sem*	1±0	1.02±0.36	0.68±0.09***	1.28±0.16	1.42±0.28**
*n*		3	3	9	3

Effects of TGFβ or LPS on the relative mRNA levels for the TGFβ receptors TβR1 and TβR2 and the Toll-like receptors TLR2 and TLR4 in murine mesangial cells (mMC) and murine glomerular endothelial cells (mGEC). Cells were incubated under control (CTRL) conditions or with the addition of TGFβ (5 ng/ml) or LPS (1 µg/ml) for 6 and 24 hours. Data for mRNA are corrected for actin expression and calculated as fold change compared to the respective untreated controls. Values represent means +/− SEM and for the number (n) of independent series of experiments indicated. Asterisks indicate: * P<0.05; ** P<0.005; *** P<0.001 as compared to the respective controls.

### The mRNA of BAMBI has a short half-life and can be stabilized by addition of cycloheximide

**Figure 6:**
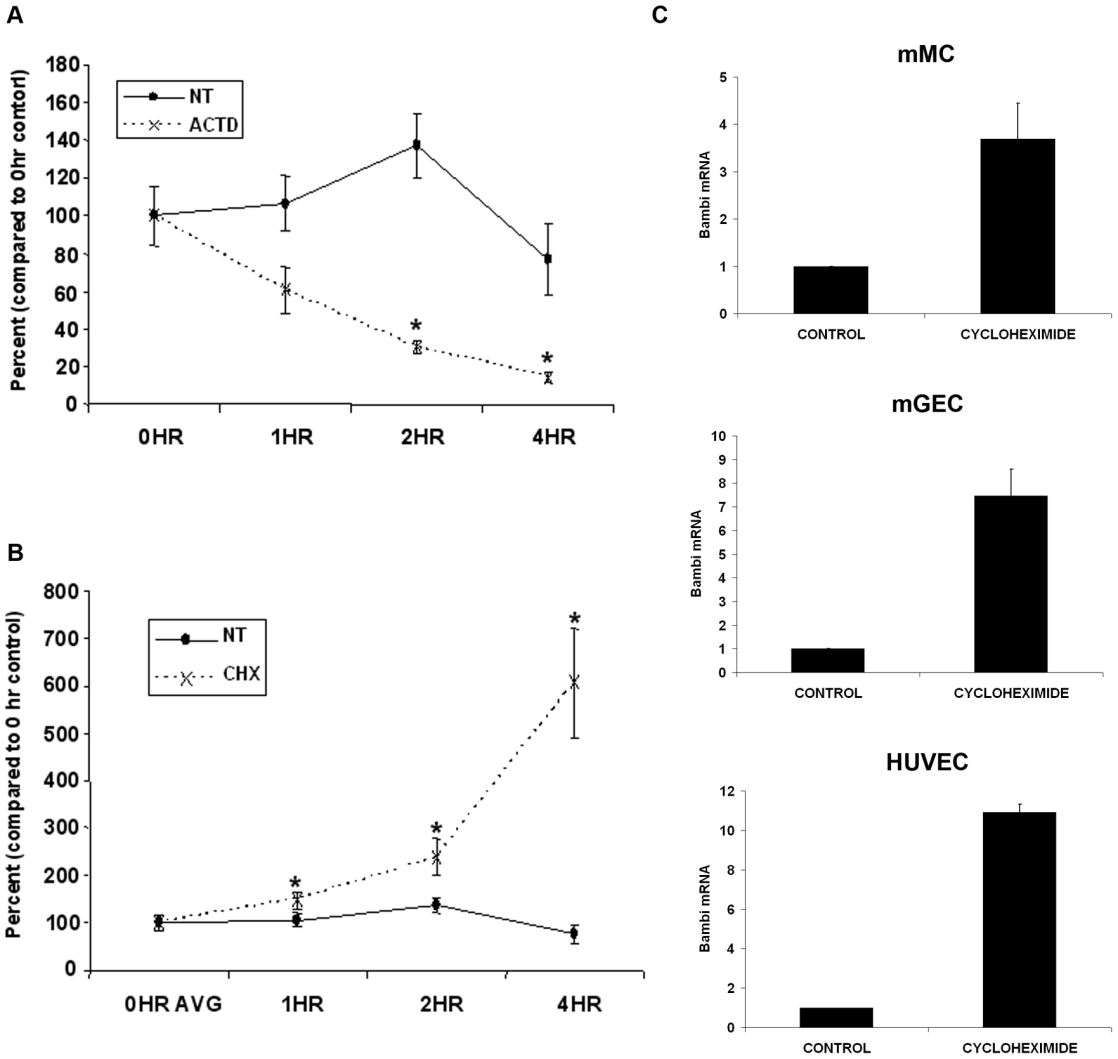
Evidence for post-transcriptional regulation of BAMBI. **6A**. Effect of actinomycin on the mRNA levels for BAMBI in murine glomerular endothelial cells (mGEC). BAMBI mRNA levels were compared in mGECs after up to 4 hours of incubation in control medium (CTRL) or in medium with addition of 5ug/ml of actinomycin to inhibit de novo mRNA synthesis. All values are expressed as percentage of the respective zero-hour time controls. Values are means +/− SEM of three series of experiments. Asterisk refer to p<0.01 for actinomycin versus controls. **6B**. Effects of cycloheximide on the mRNA levels for BAMBI in mGEC. Cells were incubated for the times indicated under control conditions or in the presence of cycloheximide (1mg/ml) prior to RNA extraction. Values for mRNA of BAMBI are expressed as percentage of the respective zero hour time controls. Values are means +/− SEM of three series of experiments. Asterisk refer to p<0.01 for cycloheximide versus controls. **6C**. Effects of 6 hours of cycloheximide treatment on the mRNA levels for BAMBI in mMCs, mGECs, and HUVECs. Values are means +/− SEM from 2–4 series of experiments for each cell type.

As the above experiments showed only minor changes in mRNA for BAMBI with LPS, we wondered if mRNA for BAMBI might also be regulated at the post-transcriptional level. The stability of some mRNAs is influenced by degradation, regulated in part by specific proteins binding to defined AU-rich base sequences (ARE elements) in the 3′ untranslated region [34]. Indeed examination of the published sequences of the 3′ untranslated sequence of the BAMBI gene for such potential sites identified two such sites in the murine and three in the human BAMBI gene sequence (Table 3). We therefore first determined the half-life of mRNA for BAMBI by experiments with actinomycin to inhibit de novo mRNA synthesis. As shown in Figure 6A the half-life of mRNA for BAMBI was in the range of 60 minutes, consistent with a rapid turnover. To examine if proteins alter the stability of the mRNA for BAMBI, we inhibited de novo protein synthesis by cycloheximide. As shown in Figure 6B cycloheximide increased mRNA for BAMBI progressively over 4 hours in mGEC. Comparable results were obtained in mMC and HUVEC (Figure 6C). Taken together these data are consistent with a post-transcriptional regulation of mRNA for BAMBI depending on de novo protein synthesis.

**Table 3:** Localization of ARE sequences in the 3′untranslated region of the BAMBI gene.

1348bp	atttattgtaaagattttttaaaatatatatatttttgtccgaaaattta	1398bp	Mouse
1663bp	atttattgtaaagatttaaaagaaatatatatattttgtctgaaatttaat	1715bp	Human

ARE sequences are underlined in the 3′ untranslated region of the murine and human BAMBI gene.

### BAMBI protein degradation is regulated by autophagy and lysosomal enzymes

**Figure 7:**
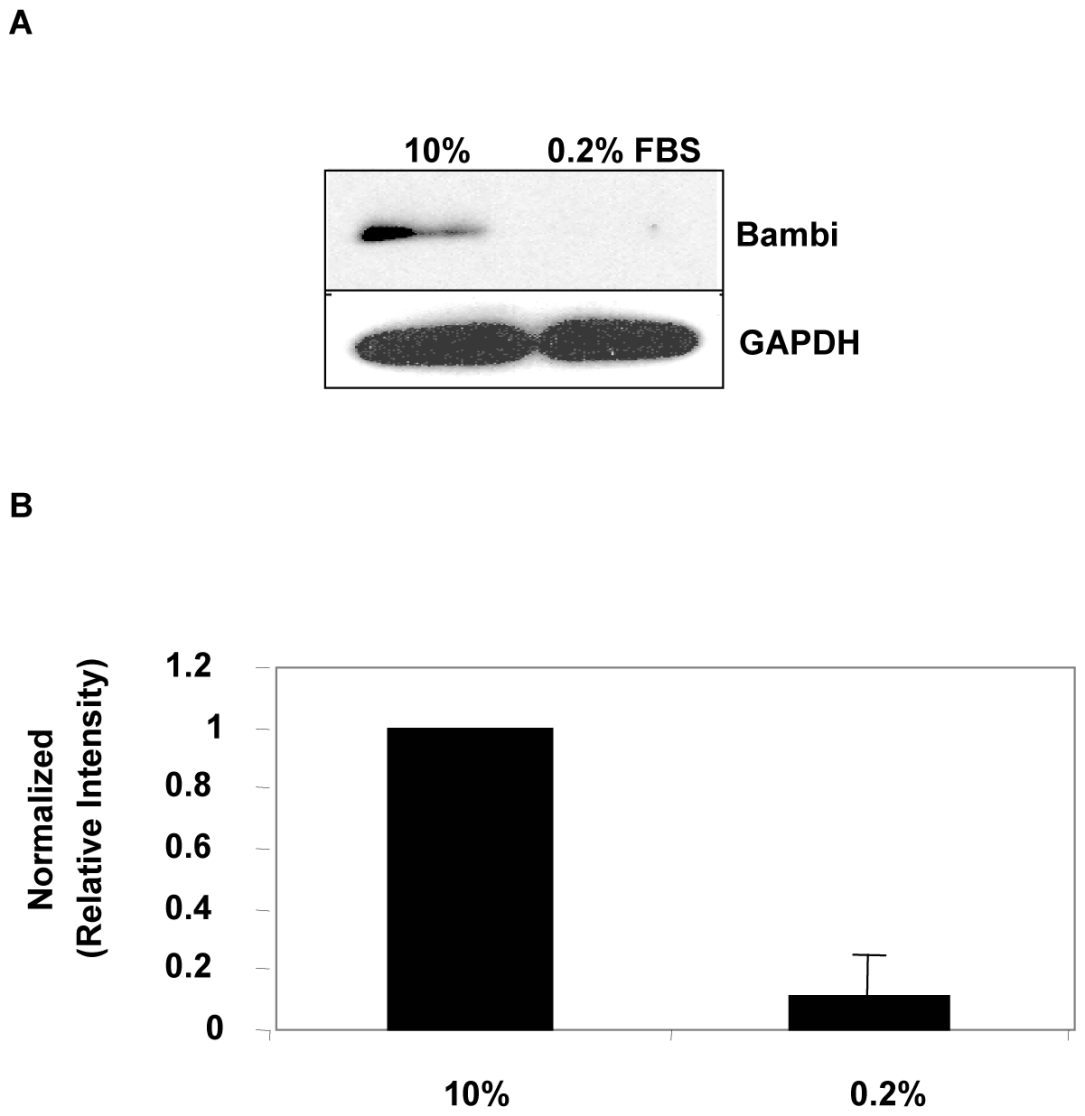
BAMBI protein levels in overexpressing HUVECs. **7A**. Representative Western blot and densitometry analysis for BAMBI in HUVECs overexpressing BAMBI (lentiviral system). Cells were cultured in 10% FBS or 0.2% FBS for 24 hours prior to protein extraction. GAPDH is shown as loading control. **7B**. For densitometry analysis, normalized relative intensities were calculated from band intensity values obtained using the NIH Image J program. Data are means +/− SEM of relative BAMBI∶GAPDH ratios (normalized to 10% FBS control cells) for two independent experiments.

Because of the short half-life of BAMBI mRNA and the post-transcriptional regulation of BAMBI we looked at BAMBI protein in the cultured cells. As the specific band for BAMBI protein was rather faint in the Western blots from mGEC and HUVEC, we used a lentiviral system to overexpress BAMBI in HUVECs. Infection of HUVECs with the lentiviral expression vector, which also contains a GFP construct, resulted in a ∼80% expression of the construct for BAMBI, as judged by GFP fluorescence (Figure S1). As to be expected this resulted in a robust overexpression of the FLAG-tagged BAMBI protein, which, due to the FLAG-tag, migrates around 29–31 kDa on the Western blot (Figure S1). Initially we examined the effect of serum starvation on the level of BAMBI protein in the HUVECs. As shown in Figure 7A, 24 hours of serum starvation resulted in almost complete disappearance of BAMBI protein on the Western blot, which was quantified in two independent experiments (Figure 7A). This occurred without changing mRNA levels for BAMBI, which were determined in parallel (results not shown).

**Figure 8:**
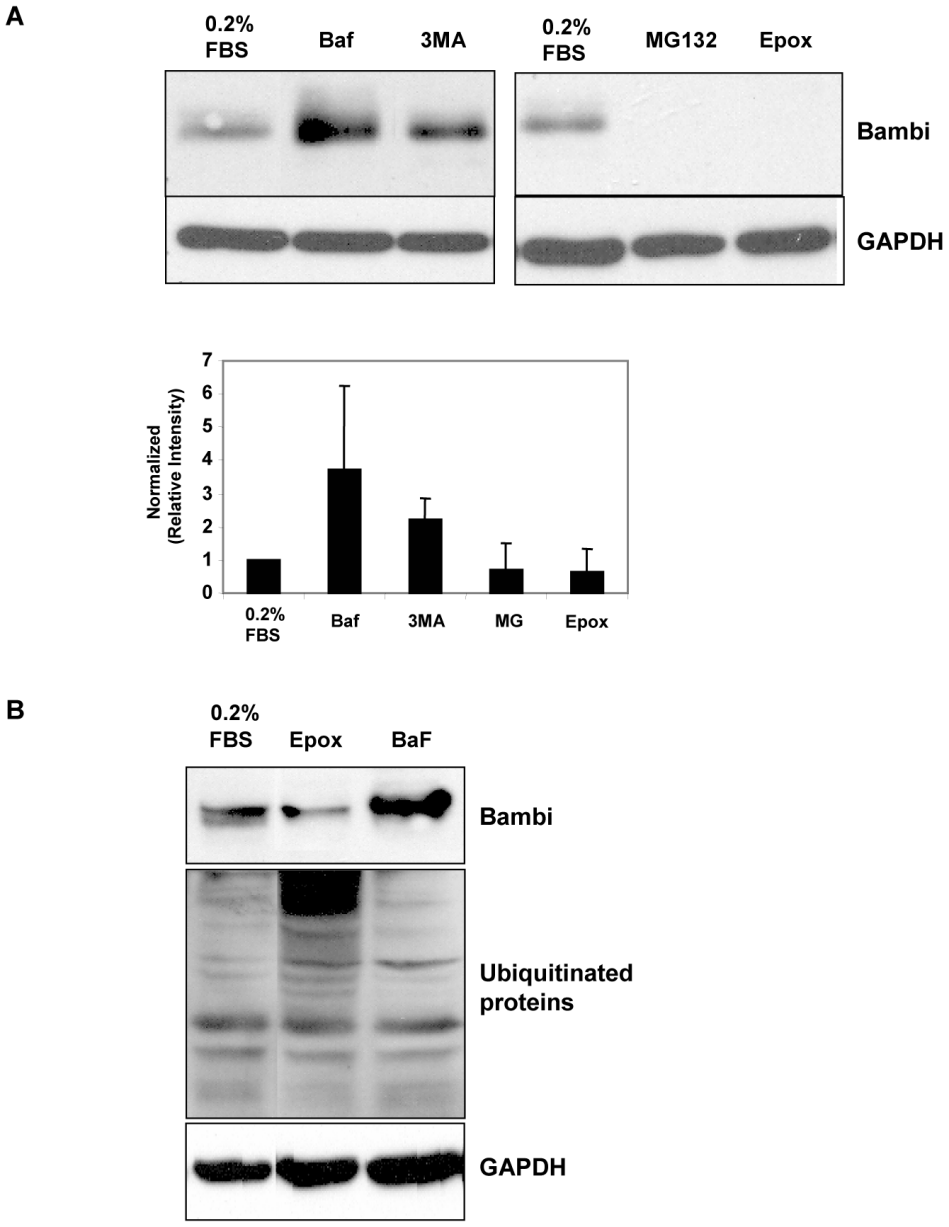
Investigation of degradation of BAMBI protein. **8A**. Western blot and densitometry for BAMBI protein in BAMBI-overexpressing HUVECs. Cells were serum-starved (0.2% FBS) for 24 hours in the absence (CTRL) or presence of bafilomycin; 3MA; MG132 or epoxomicin for 24 hours at the concentrations described in *Methods*. Normalized relative intensities were calculated from band intensity values obtained using the NIH Image J program. Data are means +/− SEM of BAMBI∶GAPDH ratios (normalized to 0.2% FBS controls) for two independent experiments. **8B**. Western blots for BAMBI, GAPDH, and ubiquitinated proteins in 293T cells infected with BAMBI-lentivirus cultured in 10% FBS and treated with bafilomycin or epoxomicin for 8 hours. After staining the Western blot for BAMBI and GAPDH, the stripped membrane was stained with an antibody for ubiquitinated proteins. While the proteasomal inhibitor epoxomicin caused a marked accumulation of ubiquitinated proteins, consistent with inhibition of their proteasomal degradation, bafilomycin did not detectably influence ubiquitinated protein degradation and retention.

**Figure 9:**
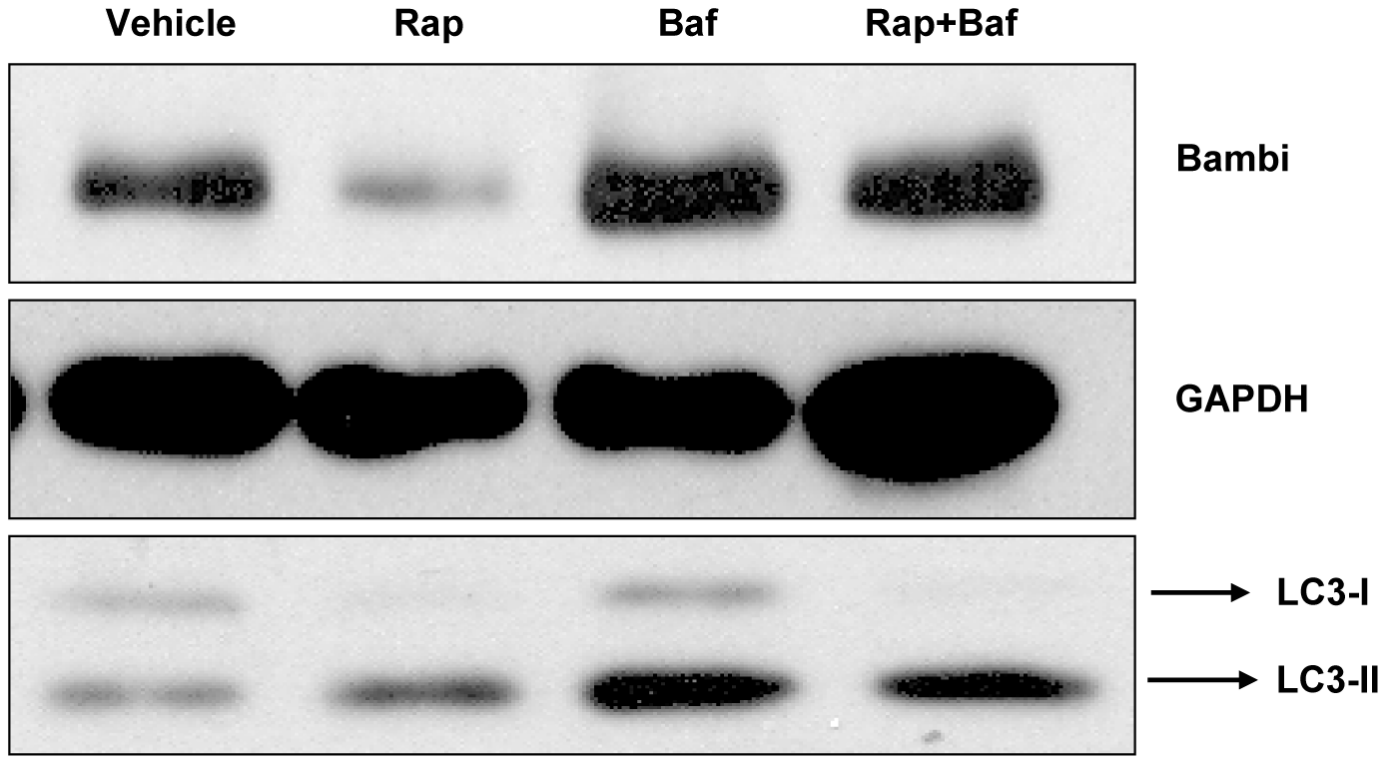
Rapamycin-induced autophagy in HUVEC. Western blot for BAMBI, GAPDH, and LC3 in BAMBI overexpressing HUVECs cultured in 10% FBS and treated with vehicle (CTRL), rapamycin, bafilomycin or bafilomycin plus rapamycin for 24 hours. Rapamycin alone reduces the BAMBI band, whereas bafilomycin alone and even in combination with rapamycin enhances it. Rapamycin causes a shift from the LC3-I to the LC3-II band, and bafilomycin alone enhances both LC3 bands. Bafilomycin does not, however, prevent the shift from LC3-I to LC3-II induced by rapamycin.

Potential pathways involved in the degradation of BAMBI protein could include proteasomal or lysosomal proteolysis or autophagy followed by autolysosomal degradation. We therefore used inhibitors of these major protein-degradation pathways. MG132 and epoxomicin were used as inhibitors of proteasomal degradation pathways [35]. Bafilomycin is an inhibitor of vacuolar proton ATPases and thereby prevents activation of lysosomal and autolysosomal proteases [36], [37]. To inhibit PI3 kinase-dependent formation of macroautophagy we used 3-methyladenine (3MA) [38], [39]. It should be noted, however, that 3MA may have additional cellular effects that can influence autophagy processes [40]. Serum starvation was used again, as it caused a marked decrease in BAMBI protein (Figure 7A and 8B). The proteasomal inhibitors MG132 and epoxomicin did not inhibit the degradation of BAMBI during serum starvation, and even increased the degradation and disappearance of BAMBI on the Western blot (Figure 8A). This may be due to enhanced lysosomal and autolysosomal degradation occurring with proteasomal inhibition, as the different proteolytic pathways seem to communicate with each other. These results were confirmed in two independent sets of experiments and quantified (Figure 8A). To demonstrate that the proteasomal inhibitor epoxomicin did in fact decrease proteasomal degradation, we determined the degree of ubiquitinated proteins, which are marked for proteasomal degradation, by reprobing the Western blot with an antibody for ubiquitinated proteins. Ubiquitinated proteins should accumulate during proteasomal inhibition, and this was in fact what we observed after epoxomicin treatment (Figure 8B). In contrast to the lack of effect of proteasomal inhibitors on BAMBI levels, inhibition of lysosomal and autolysosomal proteolysis by bafilomycin markedly enhanced the BAMBI bands on the Western blots and totally prevented the marked reduction of the BAMBI bands by serum starvation (Figure 8). As bafilomycin would also inhibit protein degradation secondary to autophagy and subsequent fusion into autolysosomes, we used 3MA as an inhibitor of early steps in macroautophagy, though 3MA may also have other effects [40]. 3MA also inhibited the degradation of BAMBI in response to serum starvation, though not to the same degree as bafilomycin (Figure 8A). As serum starvation predominantly activates macroautophagy, we also used rapamycin, which through inhibition of TOR, induces autophagy, and thereby degradation of proteins by autolysosomal pathways [41]. In HUVECs maintained at 10% FBS, rapamycin reduced BAMBI protein levels and this was associated with enhanced LC3-II formation, indicating autophagy (Figure 9). Autophagy results in lipid modification of the LC3 marker of autophagy vesicle formation, shifting it from LC3-I to LC3-II on the Western blot. The ratio of LC3-I to LC3-II can thereby serve as a monitor of ongoing autophagy [40]. Bafilomycin also prevented the rapamycin-induced decrease of BAMBI protein, but did not inhibit the formation of LC3-II (Figure 9). These results are consistent with bafilomycin inhibiting autolysosomal and lysosomal proteolysis. Comparable results were obtained in at least two to three independent sets of experiments for each experimental condition. These results further support that BAMBI can be degraded by pathways involving autophagy, in response to serum starvation or induced by rapamycin. In either case the subsequent proteolysis of BAMBI involves autolysosomal and lysosomal degradation, inhibitable by bafilomycin.

### BAMBI localization to autophagic vesicles

**Figure 10:**
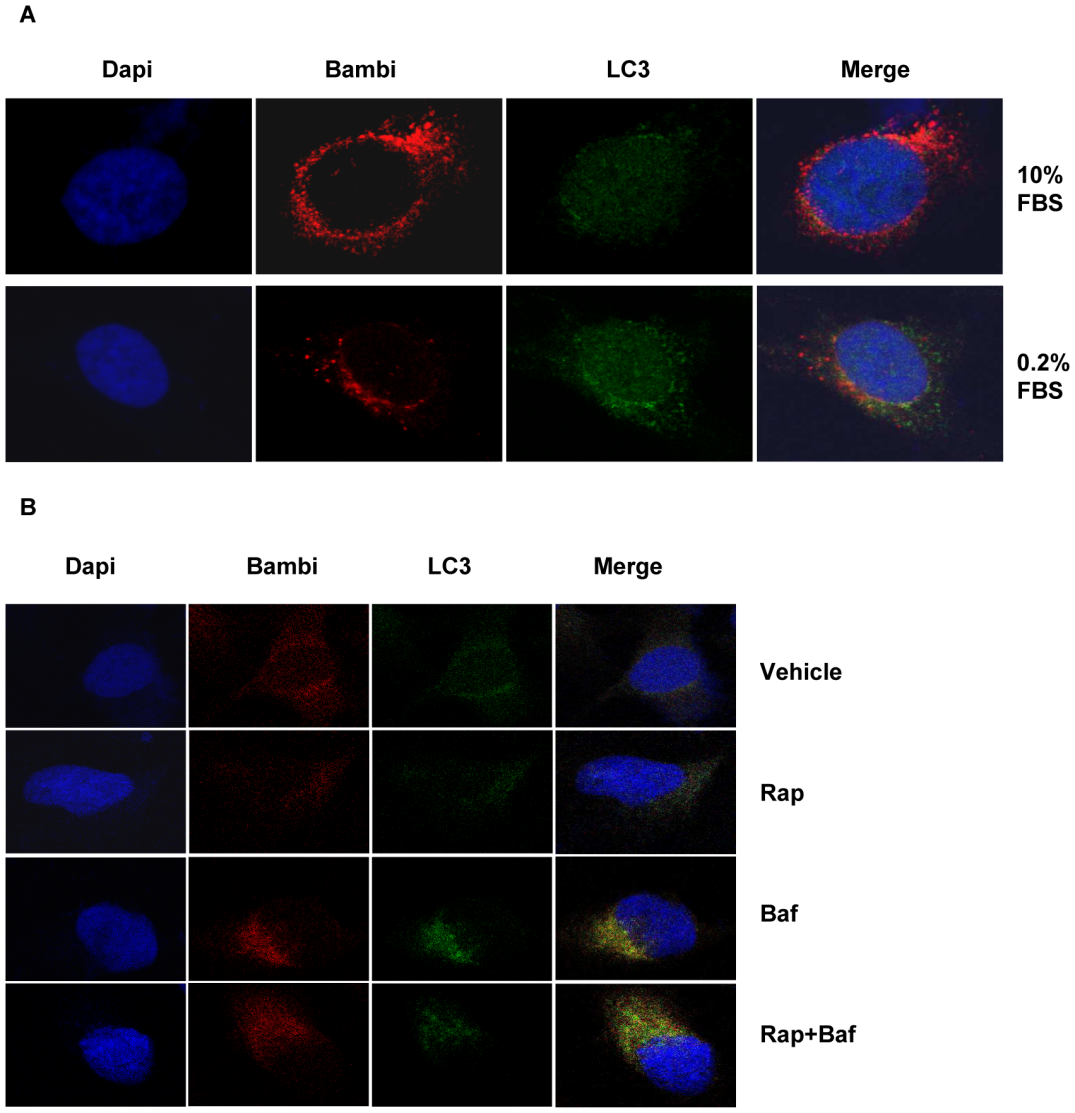
Immunofluorescence localization of BAMBI to autophagic vesicles. **10A**. Confocal images for immunofluorescence staining of BAMBI (red), LC3 (green) and DAPI (blue) in HUVECs with BAMBI overexpression. Cells were cultured in 10% or 0.2% FBS for 24 hours prior to fixation and immunofluorescence staining. Note marked perinuclear punctate staining for BAMBI and diffuse and occasional punctate staining for LC3 in cells maintained with 10% FBS with hardly any merge of signals. After 24 hours of culture in 0.2% FBS there is an overall reduction in the punctate red staining for BAMBI and a marked increase in punctate green staining for LC3, resulting in some clearly discernable colocalization and merge to an orange punctate signal in some areas. For quantification of the colocalization please refer to *Results*. **10B**. Confocal images of immunofluorescence for DAPI (blue), BAMBI (red), and LC3 (green) staining in BAMBI overexpressing HUVECs cultured in 10% FBS and treated with vehicle, rapamycin, bafilomycin or bafilomycin plus rapamycin for 24 hours. Rapamycin alone caused the diffuse LC3 staining to take on a distinct punctate appearance, which was further enhanced by bafilomycin alone or in combination with rapamycin. Rapamycin alone slightly reduced the immunofluorescence appearance of BAMBI, whereas bafilomycin alone and especially in combination with rapamycin caused a marked increase in the BAMBI-positive puncta. For quantification please refer to *Results*.

In order to localize BAMBI in HUVECs we used immunofluorescence microscopy. In non-transfected HUVECs grown in 10% FBS a faintly positive stain (red) for BAMBI could be identified with a diffuse and occasional punctate pattern (Figure S3). Staining for LC3 (green) as an autophagy marker showed a faint and diffuse cytosolic pattern in these cells (Figure S3). In order to induce autophagy, cells were treated with rapamycin, which further reduced the diffuse BAMBI staining, leaving only a few punctate BAMBI-positive spots. LC3 changed from a diffuse to mostly punctate pattern with rapamycin treatment. By superimposition some, but not all dots were positive for both BAMBI and LC3, consistent with some autophagosomal colocalization for BAMBI and LC3. Because of the weak immunofluorescence signal for BAMBI in non-transfected HUVECs, subsequent experiments were performed in HUVECs overexpressing BAMBI, as used above for the Western blot analysis. This also allowed the use of confocal microscopy for better colocalization. As shown in a picture obtained by confocal microscopy at 10% FBS, BAMBI (red staining) was seen in a perinuclear distribution and with a punctate pattern, with some larger confluent perinuclear areas, potentially representing inclusion bodies and ER-Golgi location, most likely as a consequence of the overexpression (Figure 10A). LC3 staining (green) was mostly diffuse and cytosolic with occasional puncta (Figure 10A). The occasionally punctate pattern of LC3 staining under basal conditions may indicate that the lentiviral overexpression system may induce more basal autophagy, potentially as a consequence of ER stress and an unfolded protein response [42]; [43]. By computer-assisted quantification of colocalization about 18% of the LC3-positive dots were also positive for BAMBI under basal conditions. After 24 hours of serum starvation (0.2% FBS) BAMBI staining was reduced, whereas LC3 staining shifted towards an increase of a punctate pattern. This increased the colocalization of BAMBI and LC3 in orange puncta to 40% (Figure 10A).

Rapamycin was also used to enhance autophagy and resulted in a similar change in staining pattern as noted for serum starvation (Figure 10B). The percentage of BAMBI and LC3 double-positive dots increased from 7–8% under control conditions to 12–15% with rapamycin treatment, consistent with colocalization of BAMBI to autophagy vacuoles. Incubation of the cells for 24 hours with bafilomycin to reduce lysosomal degradation caused an increase in staining of both BAMBI and LC3 with a punctate and sometimes confluent pattern even under control conditions, similar to what was observed by Western blot analysis (Figure 9). Bafilomycin alone increased the percentage of BAMBI and LC3 double staining vacuoles to 40%. Again the effect of bafilomycin was also observed during rapamycin treatment with 36% colocalization of double-positive vesicles after bafilomycin plus rapamycin treatment, as determined by computer-assisted morphometry. While this shows a robust increase in LC3 and BAMBI double-positive vesicles, it still leaves a considerable amount of BAMBI and LC3 staining in separate compartments. Thus bafilomycin inhibits both BAMBI and LC3 degradation, but only part of this occurs in LC3-positive autolysosomal compartments. Taken together the immunofluorescence results with BAMBI overexpressing HUVECs confirm those in untransfected HUVECs and are also consistent with those obtained by Western blot. Overall this indicates that BAMBI protein undergoes rapid removal by macroautophagy upon serum starvation, and by other forms of autophagy induced by rapamycin. The subsequent degradation of BAMBI occurs by autolysosomal and other lysosomal degradation inhibitable by bafilomycin, but does not appear to involve proteasomal proteolysis.

## Discussion

BAMBI has been proposed to play a role in TGFβ and Wnt signaling, and as such may markedly influence fibrosis and tumor progression. Surprisingly so far data on cell type-specific expression of BAMBI in adult mammalian tissues are not available. As both TGFβ and Wnt play major roles in renal development and disease, we examined the localization of BAMBI expression in murine and human kidneys. By immunofluorescence microscopy and by mRNA expression analysis we found that BAMBI is restricted to vascular endothelial cells predominantly of the glomerular capillaries, as well as to endothelial cells of arterial and venous blood vessels in both murine and human kidneys. TGFβ upregulated mRNA of BAMBI in glomerular endothelial cells, but contrary to expectations, LPS did not downregulate mRNA for BAMBI in murine glomerular endothelial cells or in HUVECs. Both the human and murine BAMBI genes contain AU-rich sequences in their 3′ untranslated sequences and their mRNA has a short half-life, which can be stabilized by cycloheximide. Degradation of BAMBI protein in cultured endothelial cells could be markedly diminished by inhibitors of autophagy, and of lysosomal and autolysosomal degradation, but not by proteasomal inhibitors. Further support for autophagy contributing to BAMBI degradation was obtained with rapamycin, which enhanced autophagy and BAMBI degradation, and caused colocalization of BAMBI and the autophagy marker LC3 in BAMBI overexpressing HUVECs analyzed by immunohistology and confocal microscopy. Taken together our data localize BAMBI to macro- and micro-vascular endothelial cells in the kidney and to HUVECs and indicate that intracellular BAMBI protein seems to be regulated predominantly by lysosomal and autolysosomal degradation. The endothelial localization of BAMBI may indicate novel, yet to be defined, functions of this modulator in the vascular endothelium in health and disease, and potentially even in tumor progression.

With the exception of various tumor tissues, few data are available on the organ and cell-specific expression of BAMBI. During embryonic development in the mouse, mRNA for BAMBI was localized to heart, lung, kidney, and the eye [7], all highly-vascularized organs. In adult rats, high mRNA levels were also found in kidney and lung. Immunohistological localizations for BAMBI have so far only been reported for various tumors, where the staining was assigned to the tumor cells [5], [6]. Our present data confirm the expression of BAMBI mRNA in murine and human kidney and for the first time localize BAMBI mRNA and protein to endothelial cells. Both endothelial cells of microvasculature in glomerular and to a lesser extent in peritubular capillaries as well as of arterial and venous macrovasculature stained positive for BAMBI in both mouse and human kidney. The endothelial localization was confirmed by double staining and colocalization of BAMBI with CD31 and with von Willebrand factor as markers of vascular endothelium. The specificity of the staining was supported by absence of BAMBI immunofluorescence in kidney from mice with genetic deletion of BAMBI. Furthermore, BAMBI mRNA expression was localized to vascular and glomerular endothelial cells by in situ hybridization in human kidney tissue. Thus the restriction of BAMBI expression predominantly to the endothelial cells is fully consistent with the immunohistological and in situ localization of BAMBI to endothelial cells.

The restricted expression of BAMBI mRNA to endothelial cells could also be confirmed by Western blots for BAMBI. Both human and murine kidneys showed bands reacting with the BAMBI antibody. The band at 26–28 kDa in murine tissue colocalizes with BAMBI overexpressed in 293T cells. Human BAMBI should have a molecular weight of about 28–30 kDa, as also evident on the Western blots from human tissue and cells. Furthermore both human and murine kidney showed a band at around 52 to 56 kDa. This may represent a BAMBI dimer, as also apparent in the original immunoblots on the cloning of BAMBI [1]. This dimer may be somewhat resistant to dissociation by SDS-mercaptoethanol, possibly due to disulfide bridge formation, because of the high cysteine content of BAMBI [44]. That both the 26–28 kDa and the 54 kDa bands represent BAMBI protein is supported by their absence in kidney extract from *BAMBI^−/−^* mice (Figure 1B). In agreement with the mRNA expression data, a consistent, but small band at 26–28 kDa for BAMBI was seen in protein extracts from murine glomerular endothelial cells and at 28–30 kDa in HUVECs. Taken together our immunohistological and in situ data, together with the cultured cell results, show that expression of BAMBI mRNA and protein is restricted to endothelial cells in both murine and human kidney. Furthermore HUVECs, as a general human endothelial cell, robustly express BAMBI (Figure 3A). The predominant expression of BAMBI in highly-vascularized organs, such as heart, lung, and kidney [7] may indicate that BAMBI is also mostly expressed in endothelial cells in these organs as well. The endothelial-specific expression of BAMBI may be of special interest, as the endothelium is considered to represent the major pathophysiological target for TGFβ and Wnt in vascular remodeling, and as these processes could be influenced by BAMBI.

In HepG2 hepatocellular carcinoma cells, TGFβ was reported to increase mRNA for BAMBI [33]. We therefore examined the response of glomerular endothelial cells to TGFβ and indeed observed some increase in mRNA levels for BAMBI, extending the observations in the HepG2 cells to endothelial cells. As TGFβ signaling is initiated by its binding to and activation of the receptors TβRII and TβRI, respectively, we also determined mRNA levels for the receptors, and found them to be robustly expressed in both mMCs and mGECs. In both mMCs and mGEC, addition of TGFβ upregulated mRNA for TβRI. In contrast TGFβ markedly downregulated TβRII mRNA in the glomerular endothelial cells. The differential regulation of TβRI and II is consistent with similar published observations [45], [46]. In murine hepatic stellate cells, mRNA and protein for BAMBI were reported to be decreased by LPS and this resulted in enhanced signaling of TGFβ in vitro and in increased liver fibrosis in vivo [2]. Our data for BAMBI mRNA and protein regulation by LPS in mGEC and in HUVECs differ somewhat from those reported in hepatic stellate cells [2]. LPS did not downregulate mRNA levels of BAMBI in mMCs, mGECs, or HUVECs, and did not alter the BAMBI protein level in HUVECs. We could, however confirm downregulation of BAMBI protein by LPS in cultured hepatic stellate cells, indicating different and cell type-specific levels of regulation for BAMBI. The failure of LPS to reduce mRNA levels for BAMBI in endothelial cells could not be related to the lack of the LPS receptor TLR4 or its co-factor CD14, as both of these were expressed in mGEC and HUVECs. Furthermore LPS increased mRNA levels for TLR2 and TLR4 in mGEC, consistent with in vivo data showing enhanced TLR4 expression in glomerular endothelial cells after LPS administration [47].

Thus the effect of LPS on BAMBI mRNA and protein regulation seems to be cell type-specific. Furthermore BAMBI mRNA levels in endothelial cells appear to be regulated by post-transcriptional mechanisms depending on de novo protein synthesis, as cycloheximide markedly increased mRNA levels for BAMBI in both mGECs and HUVECs (Figure 6B). This post-transcriptional process may contribute to the short half-life of BAMBI mRNA that we observed. The instability of mRNA for BAMBI may relate to the AU-rich sequences (ARE), that we identified in the published sequences of the 3′ untranslated region of both the murine and human BAMBI gene (Table 3). Such ARE have been noted to regulate mRNA at the post-transcriptional level, mostly through binding of specific proteins [34]. Recently ARE have been identified in genes involved in inflammatory responses, where they were proposed to regulate the half-life of pro-inflammatory genes [48]. A potential involvement of BAMBI in inflammation remains to be explored, but appears a reasonable possibility, given the roles of TGFβ and Wnt in inflammation.

Surprisingly, serum starvation caused a rapid disappearance of BAMBI protein in HUVECs. The major pathways for intracellular protein degradation involve the proteasome, the lysosome and autophagy. We therefore investigated the effects of inhibitors for the various pathways on BAMBI protein levels. For inhibition of proteasomal degradation we used epoxomicin and MG132. Bafilomycin was used, as it inhibits lysosomal and autolysosomal proton ATPase, and thereby the activation of acid proteases in these compartments [37]. In addition bafilomycin may also have secondary inhibitory effects on autophagy vesicle processing [36], [40], [49], [50]. 3-methyladenine (3MA) inhibits the early steps of macroautophagy in response to starvation by interfering with PI3 kinase [38], [39], but does not inhibit chaperone-mediated or alternative pathways of autophagy, and perhaps also not microautophagy [51].

We generated BAMBI overexpressing HUVECs using a lentiviral system allowing for a more reliable analysis of BAMBI protein metabolism. We used serum starvation as a means to increase protein degradation, mostly by macroautophagy. This resulted in a marked decrease in BAMBI protein. The degradation of BAMBI by serum starvation was not inhibited by the proteasomal inhibitors epoxomicin and MG132. In fact the proteasomal inhibitors caused an even more dramatic disappearance of BAMBI in the Western blot analysis. This may be due to the interdependence of the different intracellular proteolytic systems, so that proteasomal inhibitors may redirect and enhance protein degradation to lysosomal and autolysosomal pathways [52]. The activation of autophagy by serum starvation and inhibition of this pathway by 3MA was confirmed by reprobing the blots with an antibody for the autophagy protein LC3. LC3 gets modified from LC3-I to LC3-II by lipidation with phosphatidylethanolamine, resulting in a shift on the Western blot [40]. Some LC3-II was even present during the basal state, as also observed in other cell culture systems. Nonetheless the ratio of LC3-II to LC3-I can be used to evaluate changes in autophagy. Serum starvation enhanced LC3-II formation and this was decreased by 3MA. As bafilomycin inhibits degradation of LC3 in autolysosomes and may also decrease the fusion of autophagy vesicles with lysosomes [36], the bands of LC3-II were also enhanced in the presence of bafilomycin.

Rapamycin activates autophagy by inhibition of mTOR, but this form of autophagy is different from the macroautophagy induced by starvation. Induction of autophagy by rapamycin enhanced degradation of BAMBI, and, as to be expected, increased the formation of LC3-II. Taken together these results are consistent with the involvement of autophagy and autolysosomal degradation of BAMBI in HUVECs. To visualize this process, parallel experiments were performed in cell culture with immunohistological localization of BAMBI, EEA, and LC3 using fluorescence and confocal microscopy. In overexpressing HUVECs, BAMBI was seen in intracellular vesicles that only occasionally colocalized with the early endosomal marker EEA (Figure S2). Under basal conditions LC3 was distributed predominantly in a diffuse, cytosolic pattern, though LC3 positive vesicles were also visualized. This may be due to the lentiviral overexpression system for BAMBI used, resulting in some basal autophagy. This interpretation is supported by the fact that in non-transfected HUVECs, LC3 was only diffusely stained in the cytosol, without any vesicular pattern (Figure S3). Furthermore, the prominent LC3-II band already observed in the Western blots of the BAMBI overexpressing HUVECs is consistent with a considerable degree of autophagy occurring in these cells under basal conditions. Thus under basal conditions BAMBI colocalization with LC3 was observed in about 8% of BAMBI-positive vesicles. Upon serum starvation or rapamycin treatment, the total amount of BAMBI immunofluorescence decreased, but the colocalization of BAMBI and LC3 in double-positive vesicles increased from 18% to 40% with starvation and from 8% to 15% with rapamycin. Bafilomycin enhanced the vesicular BAMBI immunofluorescence both under basal conditions as well as after serum starvation or rapamycin. This could be demonstrated both by regular as well as by confocal microscopy. Taken together the results with serum starvation, rapamycin, and the inhibitor bafilomycin obtained by immunofluorescence are consistent with those obtained by Western blot analysis and support that BAMBI is degraded by processes involving autophagy and lysosomal and autolysosomal, but not proteasomal proteolysis. The lack of proteasomal inhibitors to decrease BAMBI degradation would distinguish the cellular pathways involved in the processing of BAMBI and its supposed interacting partner TβRI. In detailed studies it was reported that TβRI was localized to over 80% in intracellular vesicles, but in contrast to BAMBI, these vesicles were positive for EEA [53]. TβRI was degraded predominantly by proteasomal and to some extent also by lysosomal pathways [54]. The potential involvement of autophagy in the degradation of TβRI was not examined in the published studies. Thus different pathways may be involved in the turnover of BAMBI and TβRI, though some of the differences could also be due to the different cell types and conditions employed in our studies and those examining the turnover of TβRI.

Taken together, our studies in mouse and human kidney for the first time show endothelial cell-restricted BAMBI expression, observations also confirmed in endothelial cell culture. BAMBI expression in endothelial cells can be regulated at the transcriptional, but also at the post-transcriptional level. The post-transcriptional regulation of mRNA stability for BAMBI is probably due to the presence of AU-rich elements in the 3′ untranslated region of the BAMBI gene. Furthermore, BAMBI protein degradation is prominent, involving degradation by autophagosomal and autolysosomal processes. The proteolysis of BAMBI can be enhanced by activation of different forms of autophagy, such as macroautophagy induced by serum starvation, or by alternative forms of autophagy activated by rapamycin. Our observations of endothelial cell-specific BAMBI expression may be of considerable interest in view of the proposals that BAMBI modifies the actions of TGFβ and Wnt, and the fact that vascular endothelial cells are major targets for these cytokines. This may point towards novel roles of BAMBI for endothelial function in glomerular disease. In support of a potential role for BAMBI in kidney disease with glomerular endothelial and capillary damage and fibrosis, we observed significant induction of BAMBI mRNA expression by microarray analysis only in glomeruli isolated from kidney biopsies from 14 patients with progressive glomerular nephrosclerosis as compared to normal glomerular tissue [55]. Finally, the hypothesis that BAMBI may play a significant role in endothelial function might even extend to the reported association of BAMBI expression with tumor progression and metastasis.

## Supporting Information

Figure S1 GFP fluorescence and Western blot of abundant BAMBI protein in overexpressing HUVEC. S1A. Fluorescent and bright field images of HUVECs infected with a lentiviral vector containing a Bambi and GFP construct. Over 80% of the cells appear infected as judged by the GFP expression. S1B. Western blot for Bambi from HUVECs infected with the lentiviral vector containing only the GFP construct or the lentiviral vector containing both the GFP and Bambi construct. S1C. Immunofluorescence for Bambi staining (red) and GFP expression (green) in HUVECs infected with lentiviral vector containing only the GFP construct (GFP vector) or the lentiviral vector containing both the GFP and Bambi construct (GFP+Bambi vector). Cells infected with only the GFP vector (top row) show some staining for endogenous Bambi, and some colocalization with GFP, while those infected with the Bambi and GFP vector (bottom row) show marked overexpression of Bambi and colocalization for GFP.
(0.37 MB TIF)

Figure S2 BAMBI and EEA staining and colocalization in HUVEC. Immunofluorescence staining for Bambi (red) and early endosomal antigen (EEA1; green) in Bambi overexpressing HUVECs. Arrows indicate areas of colocalization.
(0.62 MB TIF)

Figure S3 BAMBI localization and LC3 staining in HUVECs treated with rapamycin and bafilomycin. Immunofluorescence microscopy pictures for staining of Bambi (red) and LC3 (green) in non-transfected HUVECs treated with vehicle (CTRL), rapamycin, bafilomycin, and rapamycin plus bafilomycin for 24 hours as described in Methods. In spite of the weak signals a decreased staining for Bambi and some increased granular-punctate staining pattern for LC3 is seen with rapamycin. With bafilomycin a clear increase in staining for both Bambi and LC3 is seen with colocalization to puncta and blots, which is equally apparent after bafilomycin plus rapamycin treatment.
(0.68 MB TIF)

## References

[1] Onichtchouk D, Chen YG, Dosch R, Gawantka V, Delius H (1999). Silencing of TGF-beta signalling by the pseudoreceptor BAMBI.. Nature.

[2] Seki E, De Minicis S, Osterreicher CH, Kluwe J, Osawa Y (2007). TLR4 enhances TGF-beta signaling and hepatic fibrosis.. Nat Med.

[3] Sekiya T, Adachi S, Kohu K, Yamada T, Higuchi O (2004). Identification of BMP and activin membrane-bound inhibitor (BAMBI), an inhibitor of transforming growth factor-beta signaling, as a target of the beta-catenin pathway in colorectal tumor cells.. J Biol Chem.

[4] Lin Z, Gao C, Ning Y, He X, Wu W (2008). The pseudoreceptor BMP and activin membrane-bound inhibitor positively modulates Wnt/beta-catenin signaling.. J Biol Chem.

[5] Fritzmann J, Morkel M, Besser D, Budczies J, Kosel F (2009). A colorectal cancer expression profile that includes transforming growth factor beta inhibitor BAMBI predicts metastatic potential.. Gastroenterology.

[6] Togo N, Ohwada S, Sakurai S, Toya H, Sakamoto I (2008). Prognostic significance of BMP and activin membrane-bound inhibitor in colorectal cancer.. World J Gastroenterol.

[7] Grotewold L, Plum M, Dildrop R, Peters T, Ruther U (2001). Bambi is coexpressed with Bmp-4 during mouse embryogenesis.. Mech Dev.

[8] Higashihori N, Song Y, Richman JM (2008). Expression and regulation of the decoy bone morphogenetic protein receptor BAMBI in the developing avian face.. Dev Dyn.

[9] Tsang M, Kim R, de Caestecker MP, Kudoh T, Roberts AB (2000). Zebrafish nma is involved in TGFbeta family signaling.. Genesis.

[10] Hwang I, Seo EY, Ha H (2009). Wnt/beta-catenin signaling: a novel target for therapeutic intervention of fibrotic kidney disease.. Arch Pharm Res.

[11] Chen J, Bush JO, Ovitt CE, Lan Y, Jiang R (2007). The TGF-beta pseudoreceptor gene Bambi is dispensable for mouse embryonic development and postnatal survival.. Genesis.

[12] O'Connor MN, Salles II, Cvejic A, Watkins NA, Walker A (2009). Functional genomics in zebrafish permits rapid characterization of novel platelet membrane proteins.. Blood.

[13] Merkel CE, Karner CM, Carroll TJ (2007). Molecular regulation of kidney development: is the answer blowing in the Wnt?. Pediatr Nephrol.

[14] Zerlin M, Julius MA, Kitajewski J (2008). Wnt/Frizzled signaling in angiogenesis.. Angiogenesis.

[15] Lin CL, Wang JY, Huang YT, Kuo YH, Surendran K (2006). Wnt/beta-catenin signaling modulates survival of high glucose-stressed mesangial cells.. Am Soc Nephrol.

[16] Loveland KL, Bakker M, Meehan T, Christy E, von Schonfeldt V (2003). Expression of Bambi is widespread in juvenile and adult rat tissues and is regulated in male germ cells.. Endocrinology.

[17] Satriano JA, Banas B, Luckow B, Nelson P, Schlondorff DO (1997). Regulation of RANTES and ICAM-1 expression in murine mesangial cells.. J Am Soc Nephrol.

[18] Akis N, Madaio MP (2004). Isolation, culture, and characterization of endothelial cells from mouse glomeruli.. Kidney Int.

[19] Xavier S, Niranjan T, Krick S, Zhang T, Ju W (2009). TbetaRI independently activates Smad- and CD2AP-dependent pathways in podocytes.. J Am Soc Nephrol.

[20] Demaison C, Parsley K, Brouns G, Scherr M, Battmer K (2002). High-level transduction and gene expression in hematopoietic repopulating cells using a human immunodeficiency virus type 1-based lentiviral vector containing an internal spleen focus forming virus promoter.. Hum Gene Ther.

[21] Yamawaki M, Zurbriggen A, Richard A, Vandevelde M (1993). Saponin treatment for in situ hybridization maintains good morphological preservation.. J Histochem Cytochem.

[22] Cohen CD, Frach K, Schlondorff D, Kretzler M (2002). Quantitative gene expression analysis in renal biopsies: a novel protocol for a high-throughput multicenter application.. Kidney Int.

[23] Shi S, Yu L, Chiu C, Sun Y, Chen J (2008). Podocyte-selective deletion of dicer induces proteinuria and glomerulosclerosis.. J Am Soc Nephrol.

[24] Livak KJ, Schmittgen TD (2001). Analysis of relative gene expression data using real-time quantitative PCR and the 2(−Delta delta C(T)) Method.. Methods.

[25] Bottinger EP (2007). TGF-beta in renal injury and disease.. Semin Nephrol.

[26] Bedford JJ, Leader JP, Walker RJ (2003). Aquaporin expression in normal human kidney and in renal disease.. J Am Soc Nephrol.

[27] Schmid H, Henger A, Cohen CD, Frach K, Grone HJ (2003). Gene expression profiles of podocyte-associated molecules as diagnostic markers in acquired proteinuric diseases.. J Am Soc Nephrol.

[28] Schlondorff D, Banas B (2009). The mesangial cell revisited: no cell is an island.. J Am Soc Nephrol.

[29] Mundel P, Reiser J, Zuniga Mejia BA, Pavenstadt H, Davidson GR (1997). Rearrangements of the cytoskeleton and cell contacts induce process formation during differentiation of conditionally immortalized mouse podocyte cell lines.. Exp Cell Res.

[30] Wolf G, Mueller E, Stahl RA, Ziyadeh FN (1993). Angiotensin II-induced hypertrophy of cultured murine proximal tubular cells is mediated by endogenous transforming growth factor-beta.. J Clin Invest.

[31] Haverty TP, Kelly CJ, Hines WH, Amenta PS, Watanabe M (1988). Characterization of a renal tubular epithelial cell line which secretes the autologous target antigen of autoimmune experimental interstitial nephritis.. J Cell Biol.

[32] Edgell CJ, McDonald CC, Graham JB (1983). Permanent cell line expressing human factor VIII-related antigen established by hybridization.. Proc Natl Acad Sci U S A.

[33] Sekiya T, Oda T, Matsuura K, Akiyama T (2004). Transcriptional regulation of the TGF-beta pseudoreceptor BAMBI by TGF-beta signaling.. Biochem Biophys Res Commun.

[34] Barreau C, Paillard L, Osborne HB (2005). AU-rich elements and associated factors: are there unifying principles?. Nuc Acids Res.

[35] Meng L, Mohan R, Kwok BH, Elofsson M, Sin N (1999). Epoxomicin, a potent and selective proteasome inhibitor, exhibits in vivo antiinflammatory activity.. Proc Natl Acad Sci U S A.

[36] Klionsky DJ, Elazar Z, Seglen PO, Rubinsztein DC (2008). Does bafilomycin A1 block the fusion of autophagosomes with lysosomes?. Autophagy.

[37] Yoshimori T, Yamamoto A, Moriyama Y, Futai M, Tashiro Y (1991). Bafilomycin A1, a specific inhibitor of vacuolar-type H(+)-ATPase, inhibits acidification and protein degradation in lysosomes of cultured cells.. J Biol Chem.

[38] Blommaart EF, Krause U, Schellens JP, Vreeling-Sindelarova H, Meijer AJ (1997). The phosphatidylinositol 3-kinase inhibitors wortmannin and LY294002 inhibit autophagy in isolated rat hepatocytes.. Eur J Biochem.

[39] Seglen PO, Gordon PB (1982). 3-Methyladenine: specific inhibitor of autophagic/lysosomal protein degradation in isolated rat hepatocytes.. Proc Natl Acad Sci U S A.

[40] Mizushima N (2004). Methods for monitoring autophagy.. Int J Biochem Cell Biol.

[41] Raught B, Gingras AC, Sonenberg N (2001). The target of rapamycin (TOR) proteins.. Proc Natl Acad Sci U S A.

[42] Schroder M, Kaufman RJ (2005). The mammalian unfolded protein response.. Annu Rev Biochem.

[43] Xu C, Bailly-Maitre B, Reed JC (2005). Endoplasmic reticulum stress: cell life and death decisions.. J Clin Invest.

[44] Knight C, Simmons D, Gu TT, Gluhak-Heinrich J, Pavlin D (2001). Cloning, characterization, and tissue expression pattern of mouse Nma/BAMBI during odontogenesis.. J Dent Res.

[45] Gazit D, Ebner R, Kahn AJ, Derynck R (1993). Modulation of expression and cell surface binding of members of the transforming growth factor-beta superfamily during retinoic acid-induced osteoblastic differentiation of multipotential mesenchymal cells.. Mol Endocrinol.

[46] Woodward TL, Dumont N, O'Connor-McCourt M, Turner JD, Philip A (1995). Characterization of transforming growth factor-beta growth regulatory effects and receptors on bovine mammary cells.. J Cell Physiol.

[47] El Achkar TM, Huang X, Plotkin Z, Sandoval RM, Rhodes GJ (2006). Sepsis induces changes in the expression and distribution of Toll-like receptor 4 in the rat kidney.. Am J Physiol Renal Physiol.

[48] Hao S, Baltimore D (2009). The stability of mRNA influences the temporal order of the induction of genes encoding inflammatory molecules.. Nat Immunol.

[49] Mizushima N, Levine B, Cuervo AM, Klionsky DJ (2008). Autophagy fights disease through cellular self-digestion.. Nature.

[50] Nishida Y, Arakawa S, Fujitani K, Yamaguchi H, Mizuta T (2009). Discovery of Atg5/Atg7-independent alternative macroautophagy.. Nature.

[51] Finn PF, Mesires NT, Vine M, Dice JF (2005). Effects of small molecules on chaperone-mediated autophagy.. Autophagy.

[52] Rote KV, Rechsteiner M (1983). Degradation of microinjected proteins: effects of lysosomotropic agents and inhibitors of autophagy.. J Cell Physiol.

[53] Hayes S, Chawla A, Corvera S (2002). TGF beta receptor internalization into EEA1-enriched early endosomes: role in signaling to Smad2.. J Cell Biol.

[54] Kavsak P, Rasmussen RK, Causing CG, Bonni S, Zhu H (2000). Smad7 binds to Smurf2 to form an E3 ubiquitin ligase that targets the TGF beta receptor for degradation.. Mol Cell.

[55] Neusser MA, Lindenmeyer MT, Moll AG, Segerer S, Edenhofer I (2010). Human nephrosclerosis triggers a hypoxia-related glomerulopathy.. Am J Pathol.

