# Supplementary figures and images for: BAMBI Is Expressed in Endothelial Cells and Is Regulated by Lysosomal/Autolysosomal Degradation
Source: PLoS One. 2010 Sep 24;5(9):e12995. doi: 10.1371/journal.pone.0012995 (PMC2945319; doi:10.1371/journal.pone.0012995)

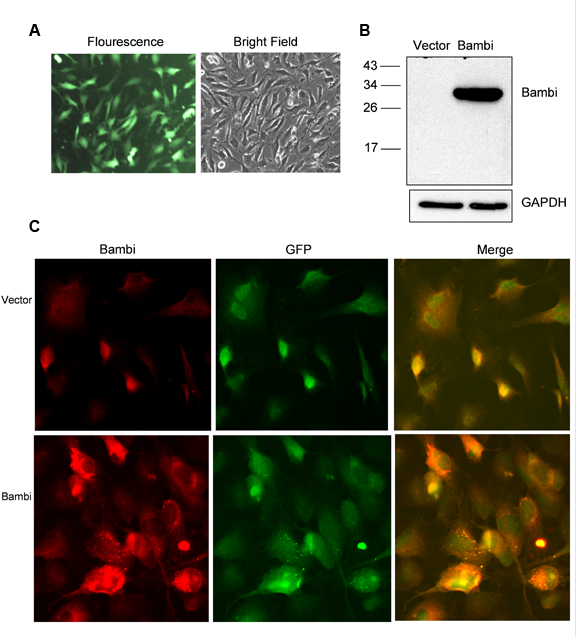

Supplement: Figure S1 — GFP fluorescence and Western blot of abundant BAMBI protein in overexpressing HUVEC. S1A. Fluorescent and bright field images of HUVECs infected with a lentiviral vector containing a Bambi and GFP construct. Over 80% of the cells appear infected as judged by the GFP expression. S1B. Western blot for Bambi from HUVECs infected with the lentiviral vector containing only the GFP construct or the lentiviral vector containing both the GFP and Bambi construct. S1C. Immunofluorescence for Bambi staining (red) and GFP expression (green) in HUVECs infected with lentiviral vector containing only the GFP construct (GFP vector) or the lentiviral vector containing both the GFP and Bambi construct (GFP+Bambi vector). Cells infected with only the GFP vector (top row) show some staining for endogenous Bambi, and some colocalization with GFP, while those infected with the Bambi and GFP vector (bottom row) show marked overexpression of Bambi and colocalization for GFP. (0.37 MB TIF) [file pone.0012995.s001.tif]

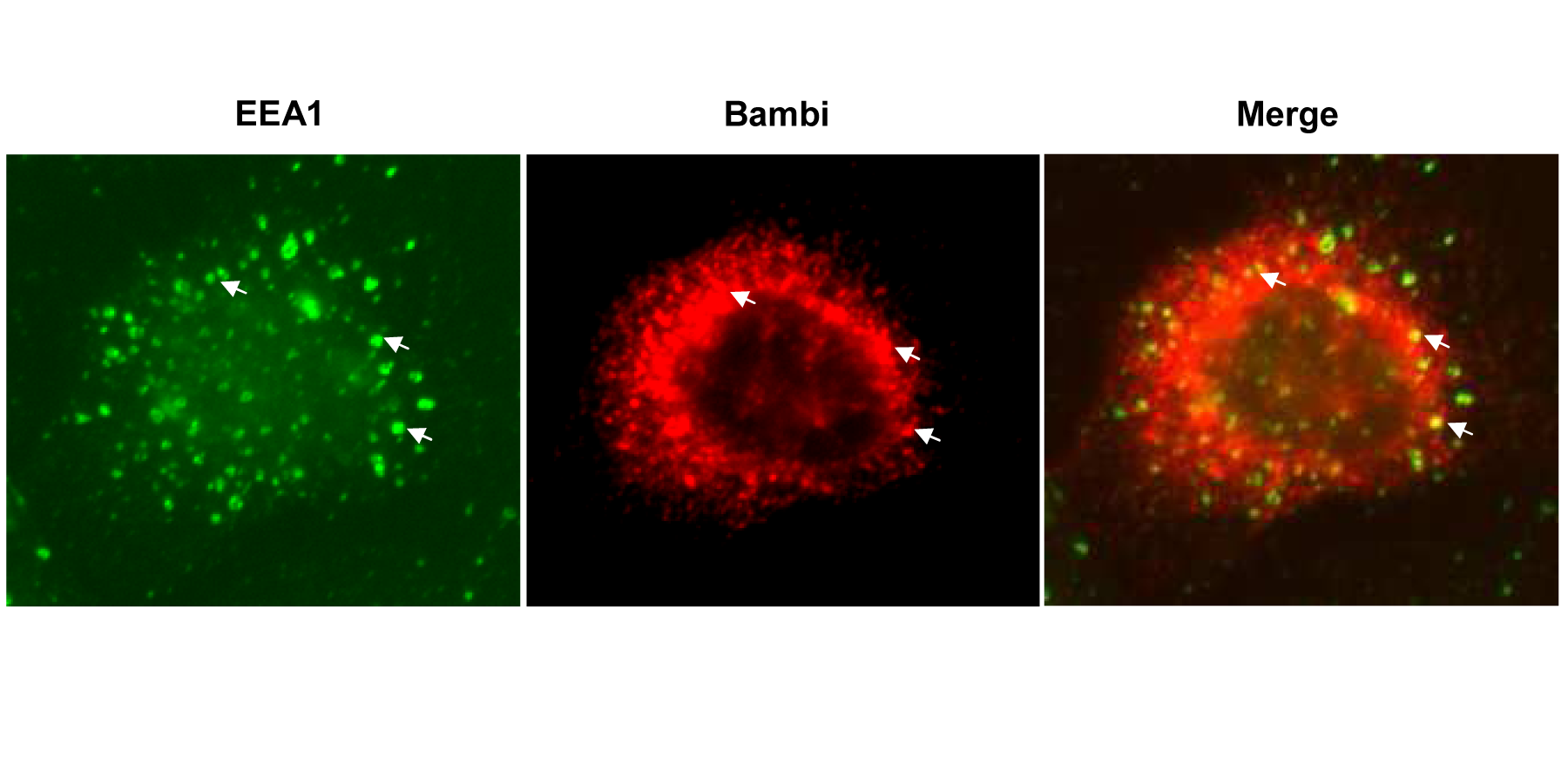

Supplement: Figure S2 — BAMBI and EEA staining and colocalization in HUVEC. Immunofluorescence staining for Bambi (red) and early endosomal antigen (EEA1; green) in Bambi overexpressing HUVECs. Arrows indicate areas of colocalization. (0.62 MB TIF) [file pone.0012995.s002.tif]

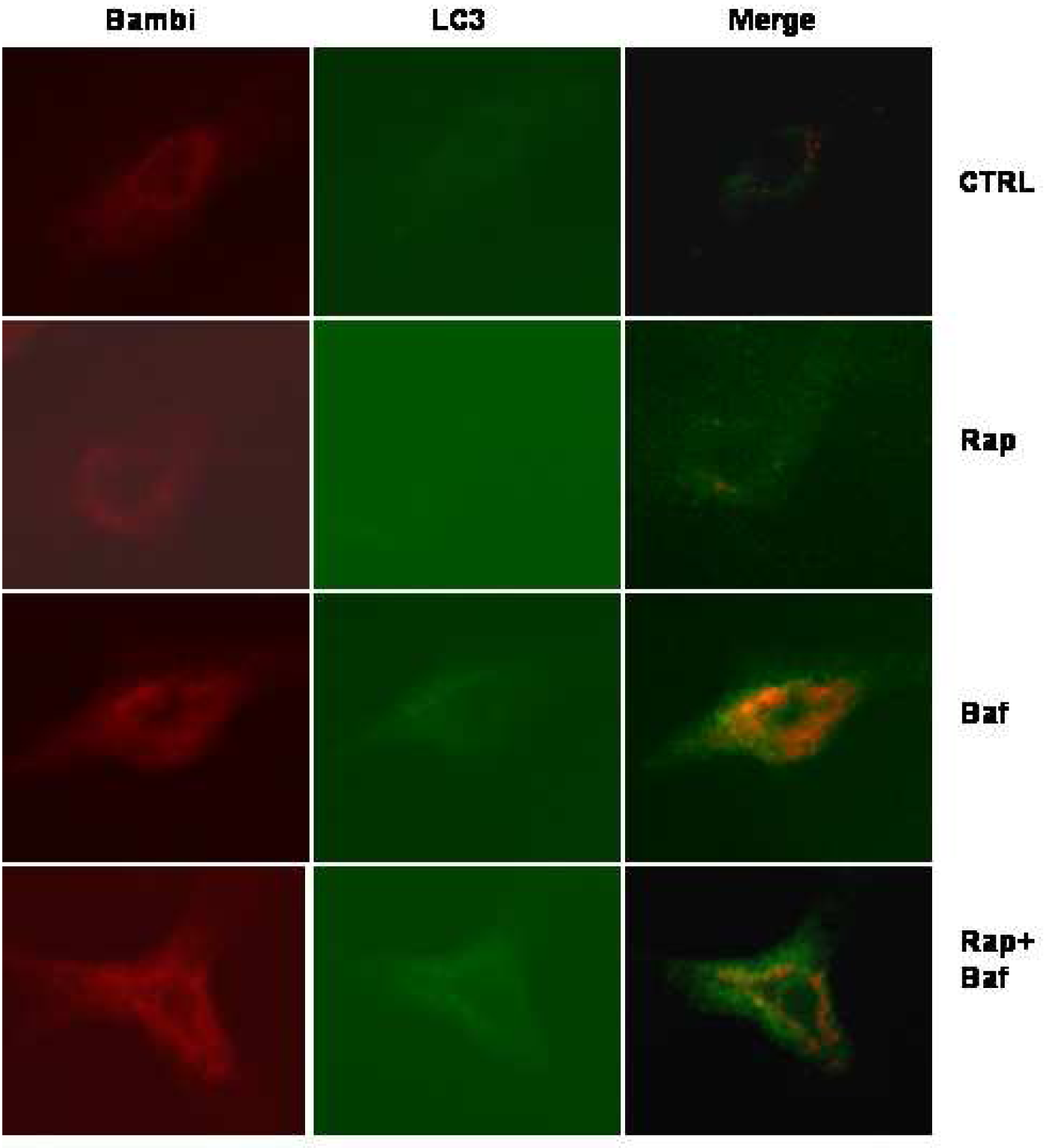

Supplement: Figure S3 — BAMBI localization and LC3 staining in HUVECs treated with rapamycin and bafilomycin. Immunofluorescence microscopy pictures for staining of Bambi (red) and LC3 (green) in non-transfected HUVECs treated with vehicle (CTRL), rapamycin, bafilomycin, and rapamycin plus bafilomycin for 24 hours as described in Methods. In spite of the weak signals a decreased staining for Bambi and some increased granular-punctate staining pattern for LC3 is seen with rapamycin. With bafilomycin a clear increase in staining for both Bambi and LC3 is seen with colocalization to puncta and blots, which is equally apparent after bafilomycin plus rapamycin treatment. (0.68 MB TIF) [file pone.0012995.s003.tif]
